# Supplementary material for: ER-residential Nogo-B accelerates NAFLD-associated HCC mediated by metabolic reprogramming of oxLDL lipophagy
Source: Nat Commun. 2019 Jul 29;10:3391. doi: 10.1038/s41467-019-11274-x (PMC6662851; doi:10.1038/s41467-019-11274-x)
Supplement: Supplementary file 1 — Supplementary Information [file 41467_2019_11274_MOESM1_ESM.pdf]

Supplementary Information

ER-residential Nogo-B accelerates NAFLD-associated HCC mediated by metabolic reprogramming of oxLDL lipophagy

Tian *et al.*

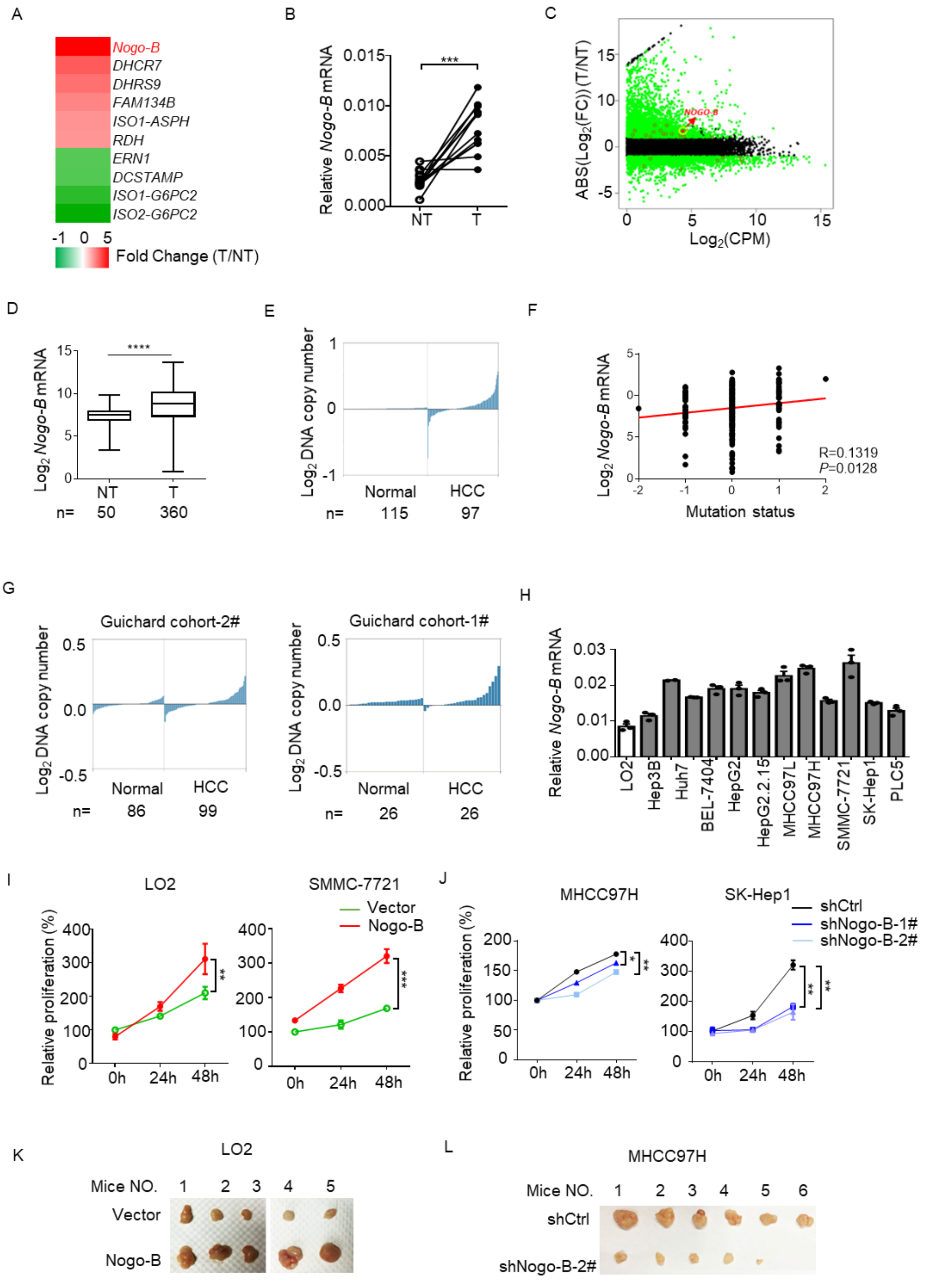

Supplementary Figure 1 *Nogo-B* promotes tumorigenicity in NAFLD-associated HCC.

(A) Heatmap presentation of the averaged fold change of 10 differentially-expressed genes in 6 paired tumors (T) and noncancerous livers (NT) from HFHC-promoted HCC mice.

(B) qRT-PCR validation of *Nogo-B* overexpression in paired tumors (T) and adjacent

noncancerous livers (NT) from HFHC-treated mice (n=11).

(C) mRNA level of genes in the TCGA database. Red circles represent ER-residential genes.

(D) mRNA level of *Nogo-B* transcript in the cBioportal website (NT, adjacent noncancerous livers; T, tumors).

(E) DNA copy number of *Nogo-B* in TCGA cohort from Oncomine website (Normal, healthy donors; HCC, tumors of HCC patients).

(F) Correlation analysis of *Nogo-B* mRNA level and DNA copy number in the HCCs in the cBioportal website (n=360; -2, deep depletion; -1, shallow depletion; 0, diploid; 1, gain; 2, amplification).

(G) DNA copy numbers of *Nogo-B* in two HCC cohorts from Oncomine website.

(H) qRT-PCR of *Nogo-B* mRNA expression in normal liver cell line LO2 and multiple HCC cell lines.

(I) MTS assay of LO2 (left) and SMMC-7721 (right) cells transfected with empty control (Vector) or Nogo-B expression plasmid (Nogo-B).

(J) MTS assay of MHCC97H and SK-Hep1 cells infected with Lenti-shCtrl (shCtrl) or Lenti-shNogo-B (shNogo-B).

(K and L) Tumor images of xenografts derived from (K) LO2 cells stably transfected with empty control or Nogo-B expression plasmid (n=5) and (L) MHCC97H cells stably transfected with control (shCtrl) or Nogo-B shRNA (shNogo-B-2#) (n=6). 5×10^6 cells were subcutaneously injected into the right and left flanks of the mice. The tumors were collected after 4 weeks.

(Data are presented as mean ± SEM of 3 independent experiments in I and J. * $p < 0.05$, ** $p < 0.01$, *** $p< 0.001$, **** $p< 0.0001$. Source data are provided as a Source Data file.)

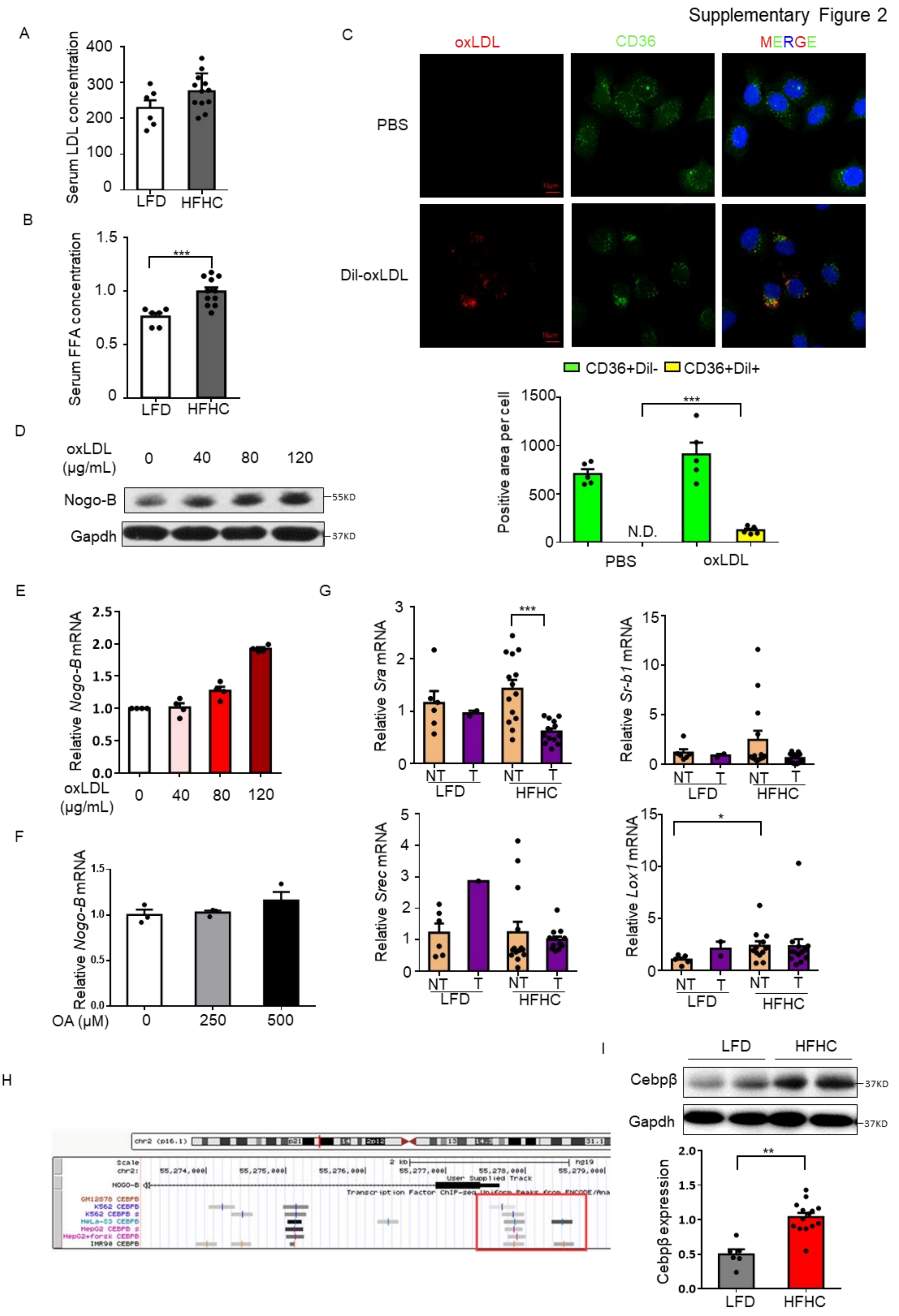

Supplementary Figure 2 Nogo-B is enhanced by the oxLDL-CD36-CEBPβ cascade.

(A and B) Serum (A) LDL and (B) FFA levels of mice fed with LFD (n=6) or HFHC (n=12).

(C) Representative immunofluorescence images (top) and quantification of positive staining areas (bottom) of LO2 cells treated with or without Dil-oxLDL (40ug/mL) for 6h.

(D) Western blot and (E) qRT-PCR analyses of Nogo-B expression in RAW264.7 cells stimulated with oxLDL at indicated dosages for 24 h.

(F) qRT-PCR analyses of Nogo-B expression in SMMC-7721 cells stimulated with oleic acid (OA) at indicated dosages for 24 h.

(G) qRT-PCR analysis of mRNA expressions of *Sra*, *Sr-b1*, *Srec* and *Lox1* in livers from LFD- (n=6) and HFHC- fed (n=14) mice.

(H) ChIP-Seq data indicates the binding site of CEBPβ to *Nogo-B* promoter.

(I) Representative Western blot images (top) and quantification (bottom) of Cebpβ expression in the livers of LFD- (n=6) and HFHC- fed (n=14) mice.

(Data are presented as mean ± SEM of 3 independent experiments in E-G.* $p < 0.05$, ** $p < 0.01$, ***$p < 0.001$. Source data are provided as a Source Data file.)

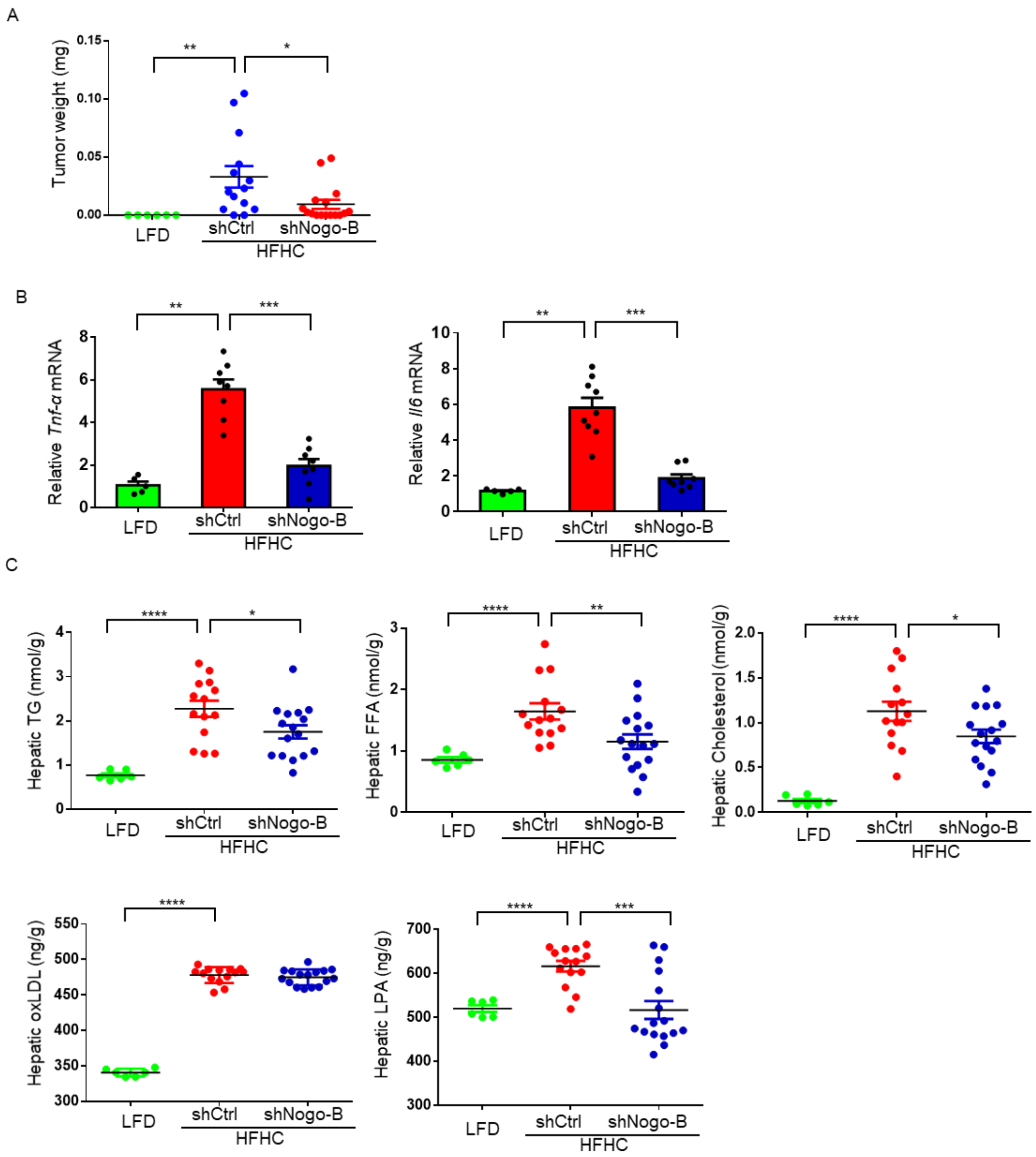

Supplementary Figure 3 Knockdown of Nogo-B inhibits NASH and HCC progression in a murine NASH-HCC model.

(A) The average tumor weight of LFD- and HFHC-fed mice described in Figure 3C.

(B) qRT-PCR of *Tnf-α* and *Il6* mRNA expressions in livers of LFD- and HFHC-fed mice described in Figure 3C.

(C) Hepatic concentrations of TG, FFA, cholesterol, LPA, and oxLDL in LFD- and HFHC-fed mice described in Figure 3C.

(Data are presented as mean ± SEM of 3 independent experiments in A-C.* $p < 0.05$, ** $p < 0.01$, ***$p < 0.001$, ****$p < 0.0001$. Source data are provided as a Source Data file.)

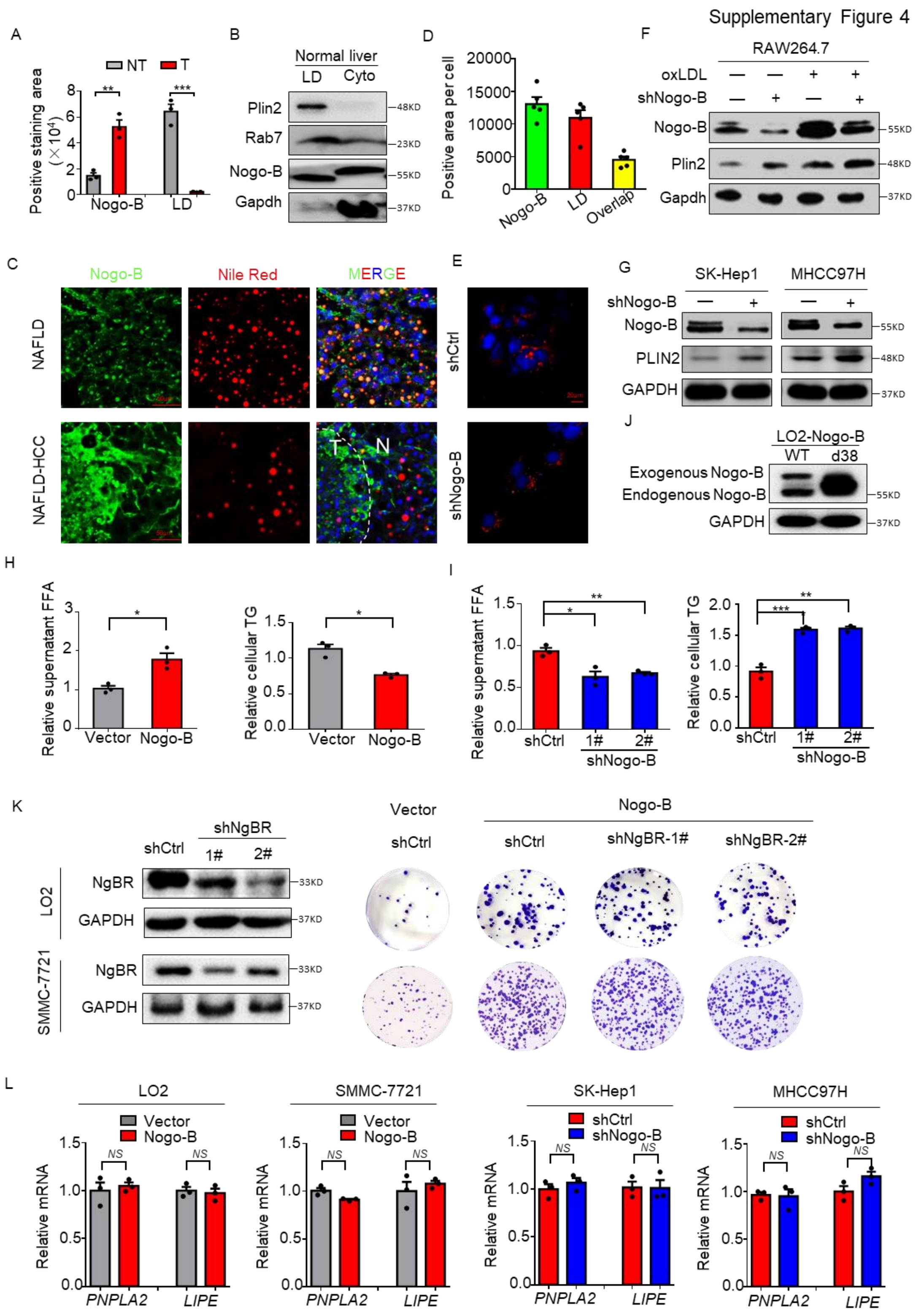

Supplementary Figure 4 Nogo-B promotes lipid droplet degradation in HCC cells.

(A) Quantification of Nogo-B and LDs staining area in murine NAFLD-associated HCCs.

(B) Western blot analysis of Nogo-B, Plin2 and Rab7 in primary isolated mouse LD and cytosol (Cyto).

(C) Co-localization of Nogo-B and LDs staining in murine NAFLD-associated HCCs.

(D) Quantification of co-localization of GFP-Nogo-B and LDs in LO2 cells transfected with Nogo-B for 48 h and incubated with oxLDL for 24 h followed by starvation.

(E) Representative LipidTOX staining of oxLDL-loaded MHCC97H cells stably transduced with shCtrl or shNogo-B without starvation.

(F) Western blot analysis of Nogo-B and Plin2 in RAW264.7 cells transfected with Nogo-B shRNA and treated with oxLDL for 24h followed by starvation.

(G) Western blot analysis of Nogo-B and PLIN2 in SK-Hep1 and MHCC97H cells stably transduced with Nogo-B shRNA and treated with oxLDL for 24h followed by starvation.

(H and I) FFA concentration in supernatant (left) and TG concentration in cell lysate (right) extracted from (H) Nogo-B-overexpressing SMMC-7721 cells and (I) SK-Hep1 cells stably transduced with shCtrl or shNogo-B and treated with oxLDL for 24 h followed by starvation.

(J) Western blot analysis of Nogo-B in LO2 cells stably transduced with Nogo-B WT or ER-motif deficient (d38) plasmid.

(K) Western Blot analysis (left) and colony formation assay (right) of stable Nogo-B expressed LO2 and SMMC-7721 cells transduced with shCtrl or shNgBRs for 14 days.

(L) qRT-PCR of *PNPLA2* and *LIPE* mRNA expressions in Nogo-B-overexpressing LO2 and SMMC-7721 cells and SK-Hep1 and MHCC97H cells stably transduced with shCtrl or shNogo-B and treated with oxLDL for 24 h followed by starvation.

(Data are presented as mean ± SEM of 3 independent experiments in A, D, H-I and L. * $p < 0.05$, ** $p < 0.01$, *** $p < 0.001$. Source data are provided as a Source Data file.)

Supplementary Figure 5

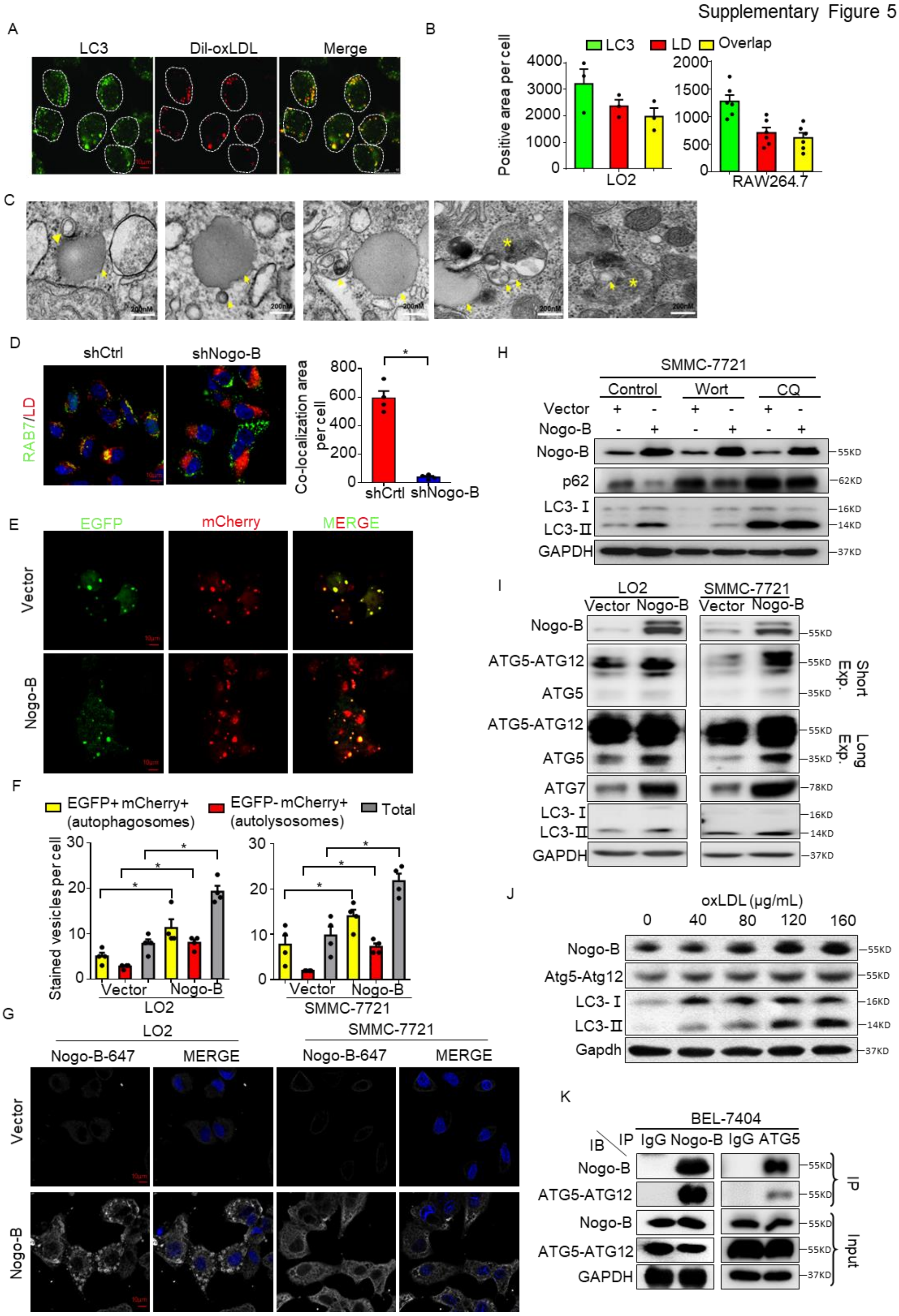

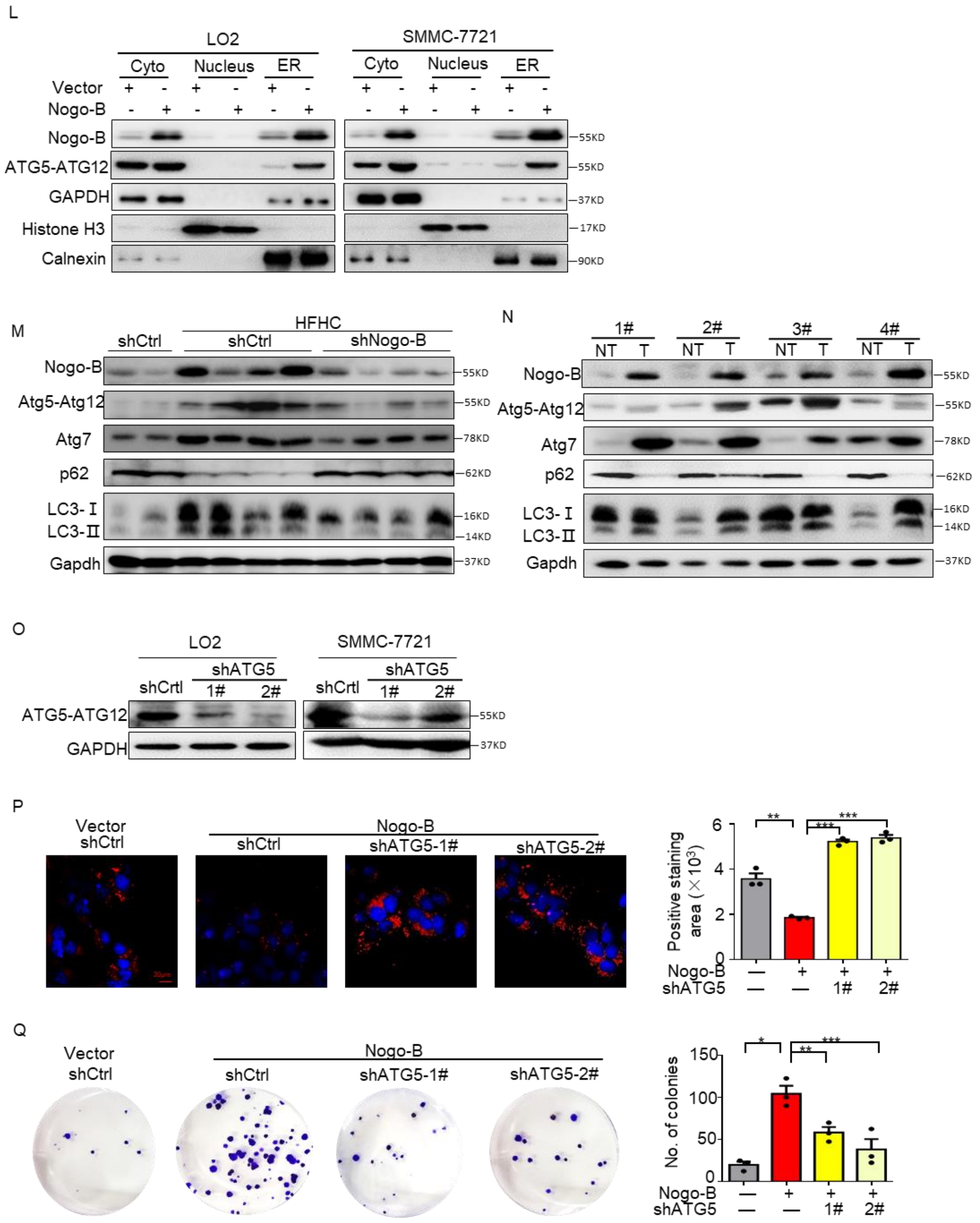

Supplementary Figure 5 Nogo-B promotes lipophagy in HCC cells.

(A) Immunofluorescence of LC3 and oxLDL in RAW264.7 cells treated with Dil-oxLDL for 24 h followed by starvation.

(B) Quantification of co-localization of LC3 and LD in LO2 and RAW264.7 cells treated with oxLDL for 24 h followed by starvation.

(C) Direct association of autophagosomes with LDs observed in electron micrographs of LO2 cells treated with oxLDL for 24h followed by starvation. Arrow heads, autophagosomes; arrows, LDs; asterisks, autolysosomes. Cells were fixed with 2.5% glutaraldehyde with Phosphate

Buffer, the ultrathin sections (70nm thick) were sectioned with microtome (Leica EM UC6) and examined by a transmission electron microscope (FEI Tecnai Spirit120kV) as described in the methods with more details.

(D) Representative immunofluorescence images (left) and quantification of co-localization areas (right) of Nogo-B and RAB7 in control or Nogo-B-knockdown MHCC97H cells treated with oxLDL for 24 h followed by starvation.

(E) Representative immunofluorescence images of control and Nogo-B-overexpressing SMMC-7721 cells transfected with mCherry-EGFP-LC3 plasmid for 48 h followed by starvation in 0.1% FBS for 12 h.

(F) Quantification of control and Nogo-B-overexpressing LO2 and SMMC-7721 cells transfected with mCherry-EGFP-LC3 plasmid for 48 h followed by starvation in 0.1% FBS for 12 h.

(G) Immunofluorescence of Nogo-B in control and Nogo-B-overexpressing LO2 and SMMC-7721 cells.

(H) Western blot analysis of Nogo-B, p62 and LC3 expression in starved control or Nogo-B-overexpressing SMMC-7721 cells treated with or without wortmannin(Wort) or chloroquine (CQ).

(I) Western blot analysis of conjugated form(ATG5-ATG12) and monomeric form of ATG5 (mono-ATG5), ATG7, and LC3 expression in starved control or Nogo-B-overexpressing LO2 and SMMC-7721 cells.

(J) Western blot analysis of Atg5 conjugated form(Atg5-Atg12), LC3-I and LC3-II expression in RAW264.7 cells stimulated with oxLDL at indicated dosages for 24 h.

(K) Co-immunoprecipitation of Nogo-B and ATG5 in BEL-7404 cells treated with oxLDL for 24h followed by starvation.

(L) Western blot analysis of Nogo-B, ATG5-ATG12, GAPDH, Histone H3 and Calnexin in different subcellular fractions.

(M) Western blot analysis of Nogo-B, Atg5-Atg12, Atg7, p62, LC3-I and LC3-II expressions in the livers of DEN-treated and, LFD- or HFHC-fed mice administered with lentivirus expressing shCtrl or shNogo-B. Mice were sacrificed at 28 weeks of age.

(N) Western Blot analysis of Nogo-B, Atg5-Atg12, Atg7, p62, LC3-I and LC3-II expression in 4 paired tumors (T) and adjacent noncancerous livers (NT) from HFHC-treated mice.

(O) Western blot analysis of ATG5-ATG12 in LO2 (left) and SMMC-7721 (right) cells transduced with control shRNA or ATG5 shRNAs.

(P) Representative LipidTOX staining (left) and quantification of positively stained areas (right) of control or stable Nogo-B-overexpressing SMMC-7721 cells transduced with indicated shRNAs and treated with oxLDL for 24 h followed by starvation.

(Q) Representative image (left) and quantification of colonies (right) of control and stable Nogo-B-overexpressing SMMC-7721 cells transduced with indicated shRNAs for 2 weeks.

(Data are presented as mean ± SEM of 3 independent experiments in B-C, E and K-L. * $p < 0.05$, ** $p < 0.01$, *** $p < 0.001$. Source data are provided as a Source Data file.)

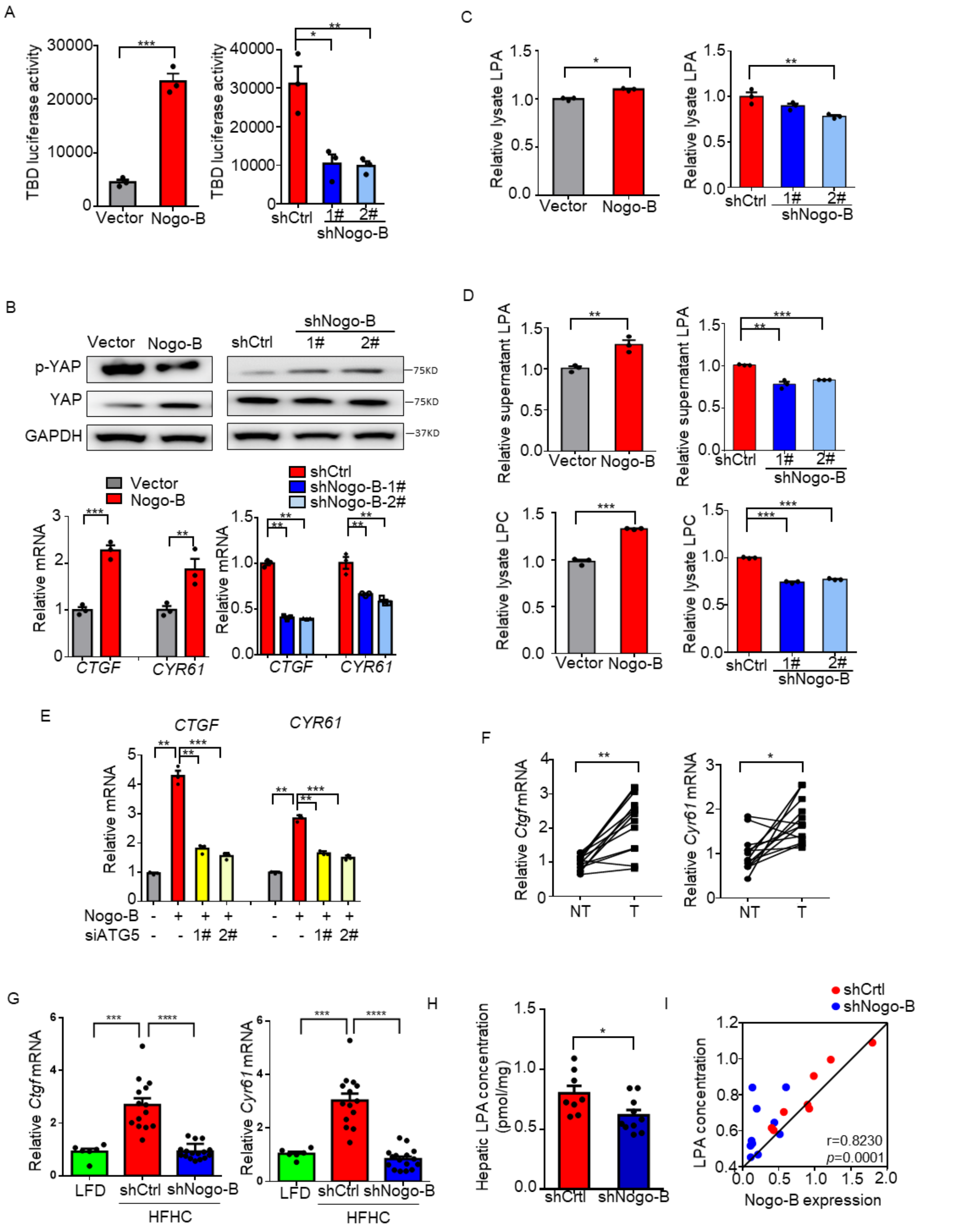

Supplementary Figure 6 Nogo-B stimulates the Hippo pathway in HCC cells.

(A) TEAD binding domain (TBD) luciferase activity of LO2 cells upon ectopic expression (left) and MHCC97H cells upon knockdown (right) of Nogo-B.

(B) Western blot (top) and qRT-PCR (bottom) analyses of YAP activity in SMMC-7721 cells upon ectopic expression (left) and SK-Hep1 cells upon knockdown (right) of Nogo-B.

(C) LPA concentrations in the cytoplasm of LO2 cells upon Nogo-B ectopic expression (left)

and MHCC97H cells upon Nogo-B knockdown (right). Stable cell lines were treated with oxLDL for 24 h followed by starvation.

(D) LPA concentrations in the supernatant (top) and LPC concentrations in the cytoplasm (bottom) of SMMC-7721 cells upon Nogo-B ectopic expression (left) and SK-Hep1 cells upon Nogo-B knockdown (right). Stable cell lines were treated with oxLDL for 24 h followed by starvation. Supernatant and cell lysate were collected for detection.

(E) qRT-PCR of *CTGF* and *CYR61* mRNA expressions in control or Nogo-B-overexpressing LO2 cells transfected with ATG5 siRNAs for 48 h and treated with oxLDL for 24 h followed by starvation.

(F) qRT-PCR of *Ctgf* and *Cyr61* mRNA expressions in paired tumors and adjacent normal tissues (NT) from HFHC-fed mice(n=13).

(G) qRT-PCR of *Ctgf* and *Cyr61* mRNA expressions in livers of LFD- and HFHC-fed mice described in Figure 3C.

(H) Total LPA concentrations in the livers from HFHC-fed mice upon administration of shCtrl (n=8) and shNogo-B (n=10).

(I) Correlation analysis of total LPA concentrations and Nogo-B expression levels in the livers from HFHC-fed mice upon administration of shCtrl (n=8) and shNogo-B (n=10).

(Data are presented as mean ± SEM of 3 independent experiments in A-E. *$p < 0.05$, ** $p < 0.01$, *** $p < 0.001$. Source data are provided as a Source Data file.)

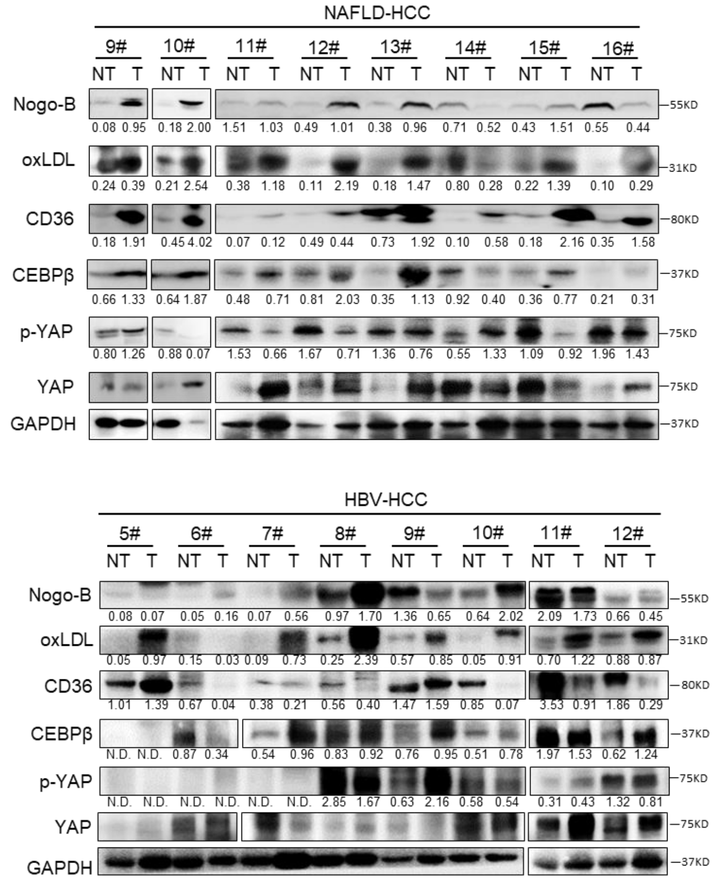

Supplementary Figure 7 Nogo-B expression positively correlates with its signaling components in primary human NAFLD-associated HCCs.

Images of Western blot analysis of upregulation of Nogo-B, oxLDL, CD36, and CEBPβ, and inhibition of YAP phosphorylation in 8 paired human NAFLD-associated HCCs (top) and 8 paired human HBV- associated HCCs (bottom) (T, tumors; NT, adjacent normal tissues).

Supplementary Figure 8

1C

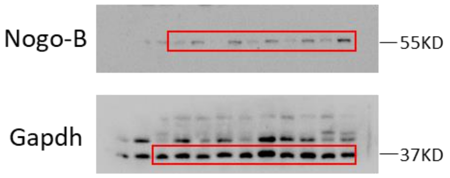

1E

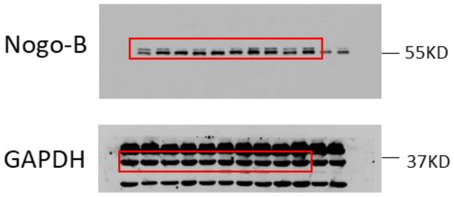

1F

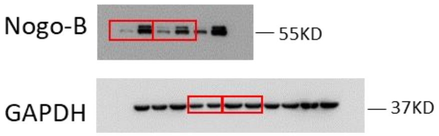

1G

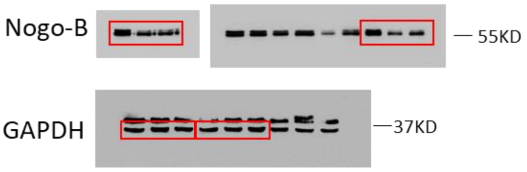

2D

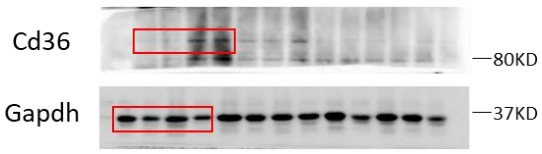
Cd36
Gapdh
80KD
37KD

2F

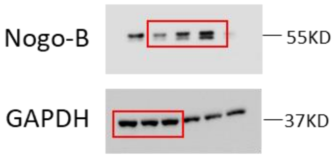
Nogo-B
GAPDH
55KD
37KD

2I

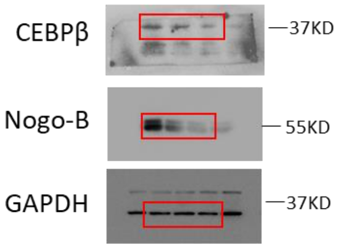
CEBPβ
Nogo-B
GAPDH
37KD
55KD
37KD

2J

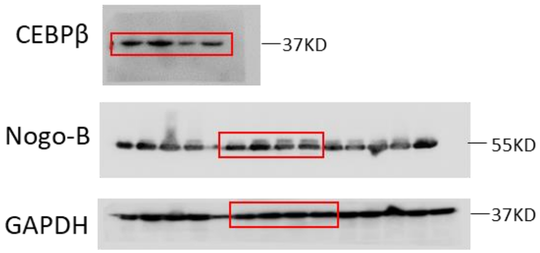
CEBPβ
Nogo-B
GAPDH
37KD
55KD
37KD

3B

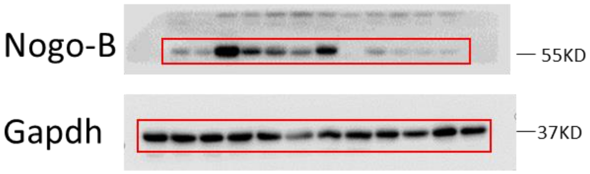
Nogo-B
Gapdh
55KD
37KD

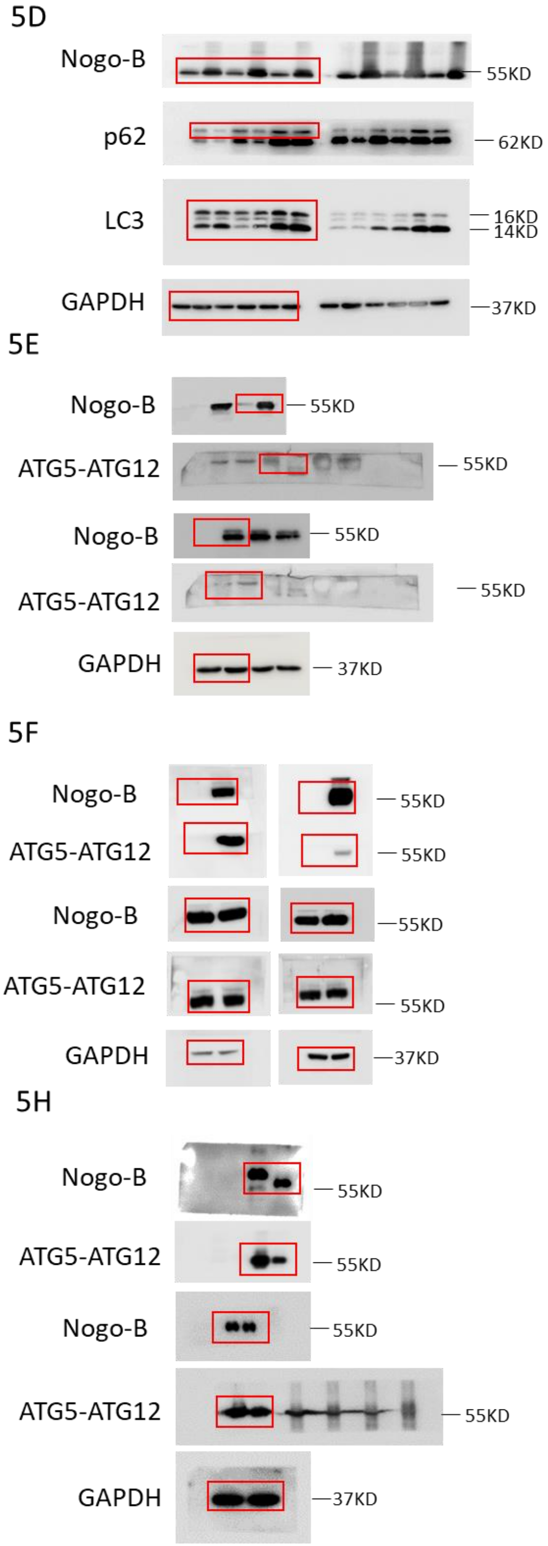
5D
Nogo-B
55KD
p62
62KD
LC3
16KD
14KD
GAPDH
37KD
5E
Nogo-B
55KD
ATG5-ATG12
55KD
Nogo-B
55KD
ATG5-ATG12
55KD
GAPDH
37KD
5F
Nogo-B
55KD
ATG5-ATG12
55KD
Nogo-B
55KD
ATG5-ATG12
55KD
GAPDH
37KD
5H
Nogo-B
55KD
ATG5-ATG12
55KD
Nogo-B
55KD
ATG5-ATG12
55KD
GAPDH
37KD

6C

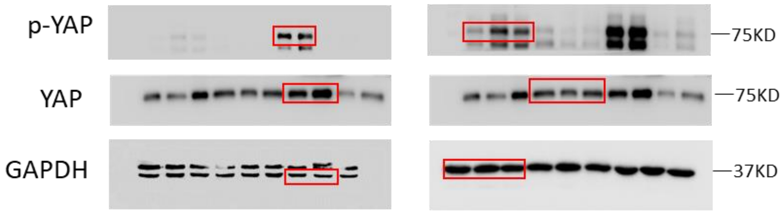
p-YAP
YAP
GAPDH
75KD
75KD
37KD

6E

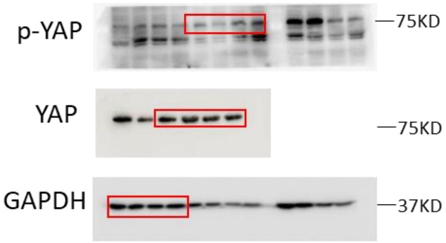
p-YAP
YAP
GAPDH
75KD
75KD
37KD

6G

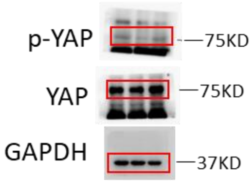
p-YAP
YAP
GAPDH
75KD
75KD
37KD

6H

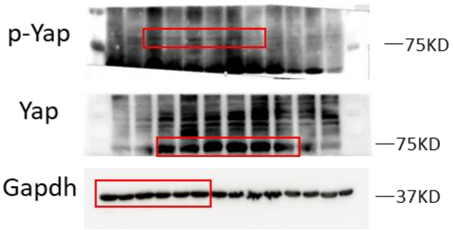
p-Yap
Yap
Gapdh
75KD
75KD
37KD

6I

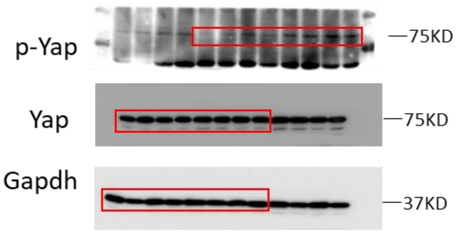
p-Yap
Yap
Gapdh
75KD
75KD
37KD

S2D

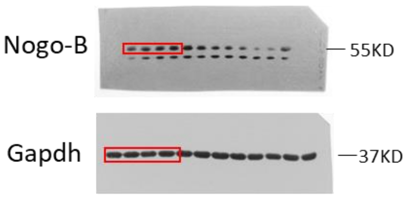

S2I

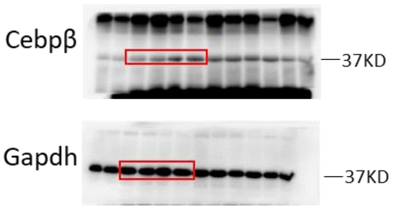

S4B

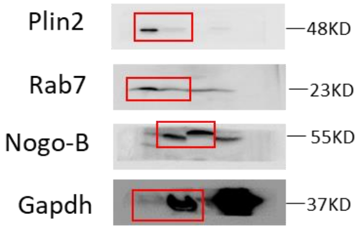

S4F

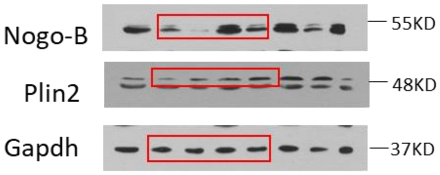

S4G

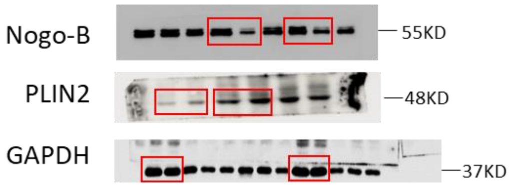

S4J

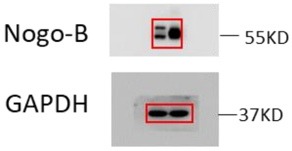

S4K

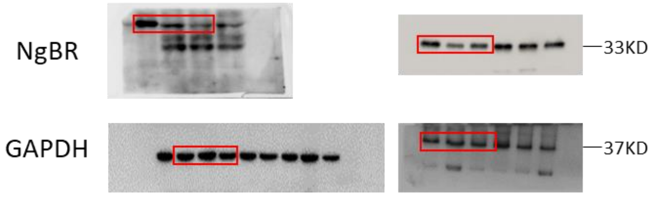

Supplemental Figure 8

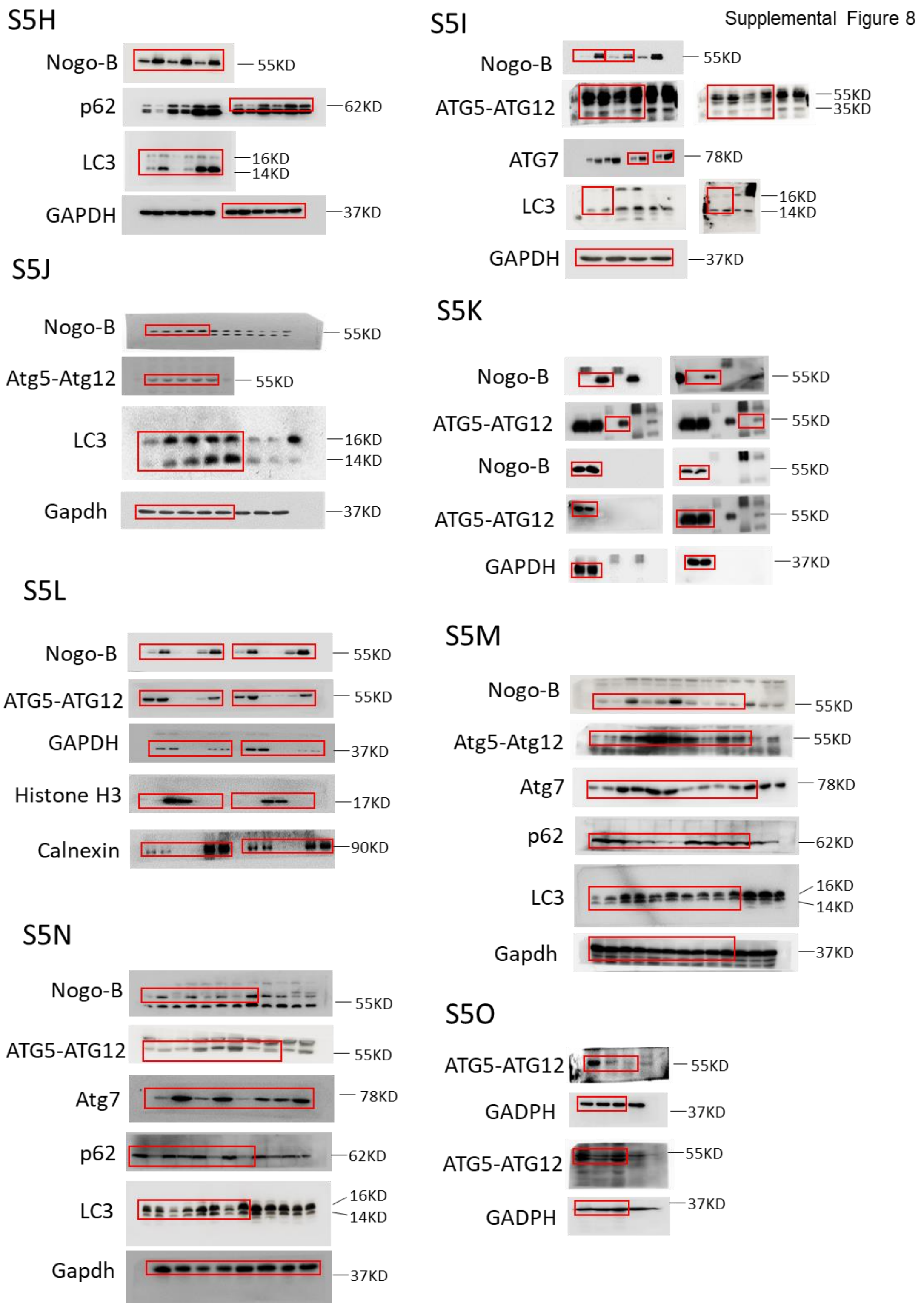

S6B

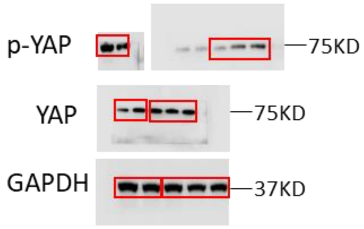

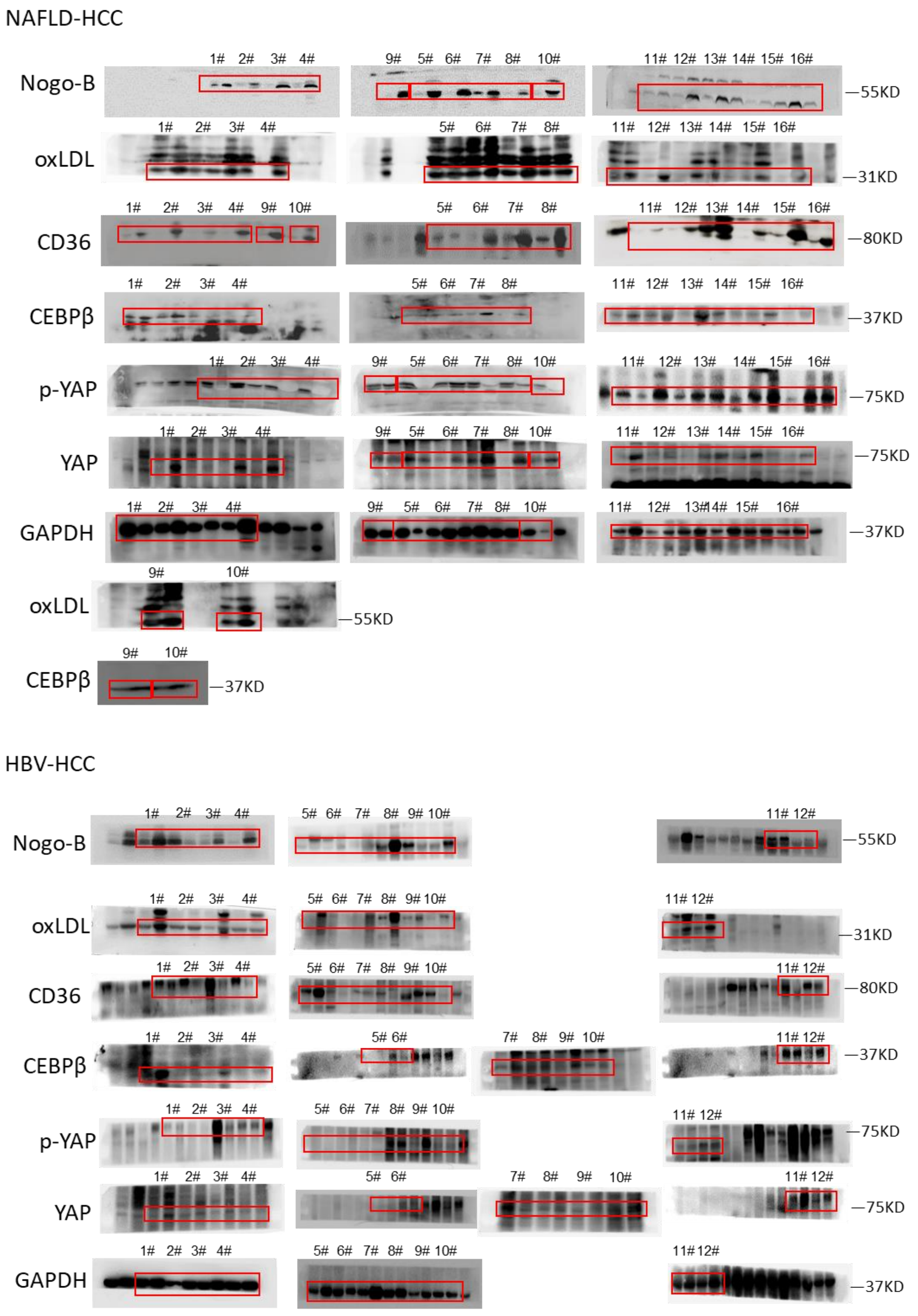

Supplementary Figure 8 Uncut full western blots gels.

Images of uncut full western blots gels.

Supplementary Table 1. Gene expression profiling of 103 ER residential gene transcripts in murine HFHC-promoted HCC models.

Gene name	Fold change (T/NT)				Gene name	Fold Change (T/NT)			
	1#	2#	3#	Mean		1#	2#	3#	Mean
Nogo-B	3.43	3.05	4.01	**3.50**	*FITM1*	1.22	0.66	1.24	**1.04**
DHCR7	2.11	2.76	2.95	**2.61**	*EDEM1*	0.78	1.28	1.02	**1.03**
DHRS9	2.60	2.26	2.36	**2.41**	*XBP1*	0.85	1.27	0.92	**1.01**
FAM134B	1.74	1.88	2.98	**2.20**	*PIGU*	0.78	1.00	1.26	**1.01**
ISO1-ASPH	1.71	1.84	2.64	**2.07**	*ESYT1*	0.84	0.89	1.28	**1.01**
RDH	1.81	2.25	2.07	**2.04**	*EXT1*	0.72	0.97	1.31	**1.00**
DERL1	1.20	2.39	1.89	**1.83**	*ISO1-XBP1*	1.13	1.77	0.06	**0.99**
ASPH	0.96	2.26	1.51	**1.58**	*ISO2-ASPH*	1.05	1.67	0.22	**0.98**
ABCB9	1.20	1.41	2.08	**1.57**	*SARAF*	1.15	1.29	0.49	**0.98**
TBL2	0.94	1.39	2.20	**1.51**	*G6PC2*	0.77	1.19	0.96	**0.97**
SAMD8	1.00	1.23	2.07	**1.43**	*ISO2-ERN1*	1.35	1.27	0.27	**0.96**
LBR	0.83	1.88	1.43	**1.38**	*ISO2-ESYT1*	1.23	0.92	0.73	**0.96**
BSCL2	1.16	1.08	1.88	**1.37**	*SYVN1*	0.84	2.03	0.00	**0.96**
AMFR	1.26	1.15	1.65	**1.35**	*PIGK*	0.92	1.14	0.74	**0.93**
RRBP1	0.92	2.19	0.93	**1.35**	*TAP1*	0.67	1.02	1.06	**0.92**
CREB3	1.02	1.21	1.80	**1.34**	*ISO1-ESYT1*	1.04	0.63	1.07	**0.91**
DERL2	1.43	1.08	1.43	**1.32**	*ISO5-PORCN*	0.96	1.03	0.73	**0.91**
ISO2-XBP1	1.53	1.64	0.73	**1.30**	*FITM2*	0.84	0.58	1.29	**0.90**
DGAT2	1.38	1.40	1.12	**1.30**	*TAP2*	1.19	1.21	0.28	**0.89**
AUP1	1.04	1.33	1.49	**1.29**	*KTN1*	0.88	1.07	0.71	**0.89**
FKBP8	1.17	1.11	1.54	**1.27**	*RTN1*	0.48	0.46	1.65	**0.86**
BFAR	1.08	1.24	1.47	**1.26**	*SACM1L*	0.83	1.09	0.64	**0.85**
CLN3	0.50	1.75	1.48	**1.24**	*PIGT*	0.93	1.05	0.57	**0.85**
ATF6	0.80	1.44	1.48	**1.24**	*RTN2*	0.62	0.76	1.16	**0.85**
ANKLE2	1.23	1.18	1.30	**1.24**	*LRIT1*	0.65	0.75	1.11	**0.83**
PORCN	0.80	1.45	1.45	**1.23**	*SGPL1*	1.04	1.11	0.28	**0.81**
DPAGT1	1.07	1.32	1.28	**1.23**	*ISO2-SYVN1*	0.71	1.46	0.27	**0.81**
TM7SF2	1.68	0.94	1.03	**1.22**	*ISO1-SYVN1*	0.87	1.35	0.17	**0.79**
DOLPP1	1.35	0.99	1.26	**1.20**	*ISO1-ERN1*	1.18	1.15	0.02	**0.78**
DPM3	0.97	1.00	1.61	**1.19**	*SLC27A2*	1.00	0.69	0.66	**0.78**
ISO1-BFAR	2.11	1.12	0.20	**1.14**	*EMC6*	0.79	0.89	0.66	**0.78**
DOLK	1.26	0.88	1.28	**1.14**	*LRMP*	0.55	0.47	1.29	**0.77**
ISO1-PORCN	1.41	1.08	0.91	**1.13**	*ELOVL6*	0.96	0.71	0.64	**0.77**
PIGG	0.77	1.15	1.47	**1.13**	*TECR*	0.84	1.20	0.20	**0.75**
ATF6B	0.90	1.31	1.14	**1.12**	*ISO2-BFAR*	1.05	0.94	0.22	**0.74**
WFS1	0.73	1.48	1.13	**1.12**	*UBXN8*	0.82	0.80	0.57	**0.73**
RHBDD1	0.85	1.34	1.12	**1.10**	*DERL3*	0.57	0.90	0.68	**0.72**
ACER3	0.87	1.24	1.18	**1.10**	*SPCS1*	0.81	1.30	0.02	**0.71**
BNIP1	0.87	1.10	1.29	**1.09**	*G6PC3*	0.81	0.85	0.43	**0.70**

HSPA5	0.77	2.48	0.01	**1.09**	*G6PC*	0.81	0.27	0.95	**0.68**
DPM2	0.80	1.09	1.37	**1.09**	*SLC27A5*	1.02	0.97	0.00	**0.66**
TEX261	1.07	0.89	1.28	**1.08**	*SLC37A4*	1.03	0.92	0.00	**0.65**
STIM1	1.76	1.35	0.11	**1.07**	*ERN1*	0.63	0.41	0.21	**0.42**
ELOVL1	1.00	1.02	1.18	**1.07**	*DCSTAMP*	0.35	0.54	0.36	**0.42**
RCE1	0.49	1.44	1.22	**1.05**	*ISO1-G6PC2*	0.18	0.24	0.42	**0.28**
SEC61A1	0.86	2.05	0.24	**1.05**	*ISO2-G6PC2*	0.10	0.13	0.19	**0.14**

Significantly overexpressed genes are marked in red; Significantly low-expressed genes are marked in green.

Supplementary Table 2. Gene expression profiling of 85 Fatty liver gene expressions in murine HFHC-promoted HCC models.

Gene name	NT					T					Fold change
	1#	2#	3#	4#	Mean	1#	2#	3#	4#	Mean	
Abca1	0.08	0.09	0.22	0.13	**0.13**	0.08	0.09	0.12	0.13	**0.11**	**0.80**
Abcg1	0.00	0.00	0.00	0.00	**0.00**	0.00	0.00	0.00	0.00	**0.00**	**1.16**
Acaca	0.06	0.08	0.14	0.09	**0.09**	0.06	0.05	0.05	0.07	**0.06**	**0.61**
Acadl	0.15	0.23	0.49	0.33	**0.30**	0.41	0.33	0.38	0.36	**0.37**	**1.24**
Acly	0.61	0.44	0.74	0.80	**0.65**	0.51	0.44	0.33	0.59	**0.47**	**0.72**
Acox1	1.23	1.83	1.83	1.61	**1.62**	2.60	1.35	1.30	1.71	**1.74**	**1.07**
Acsl5	0.47	0.45	0.60	0.52	**0.51**	0.57	0.39	0.25	0.57	**0.45**	**0.87**
Acsm3	0.13	0.21	0.30	0.16	**0.20**	0.23	0.23	0.10	0.10	**0.17**	**0.84**
Adipor1	0.10	0.12	0.20	0.16	**0.14**	0.14	0.11	0.14	0.14	**0.13**	**0.92**
Adipor2	0.04	0.05	0.08	0.08	**0.06**	0.07	0.05	0.05	0.09	**0.06**	**1.10**
Akt1	0.02	0.02	0.04	0.03	**0.03**	0.02	0.02	0.03	0.03	**0.03**	**0.98**
Apoa1	8.57	14.79	26.46	16.63	**16.62**	13.45	10.15	17.57	11.82	**13.25**	**0.80**
Apob	5.35	6.16	9.54	8.26	**7.33**	7.17	8.51	7.89	6.96	**7.63**	**1.04**
Apoc3	4.94	5.84	10.32	9.53	**7.66**	7.40	8.09	9.18	8.18	**8.21**	**1.07**
Apoe	10.06	14.53	27.36	19.36	**17.83**	19.43	13.85	23.28	13.47	**17.51**	**0.98**
Atp5c1	0.29	0.39	0.70	0.53	**0.48**	0.51	0.53	0.42	0.40	**0.46**	**0.97**
Casp3	0.02	0.02	0.04	0.04	**0.03**	0.02	0.02	0.02	0.03	**0.02**	**0.66**
Cd36	0.01	0.04	0.05	0.02	**0.03**	0.23	0.17	0.05	0.12	**0.14**	**4.86**
Cebpb	0.04	0.07	0.08	0.09	**0.07**	0.15	0.17	0.12	0.15	**0.15**	**2.00**
Cnbp	0.23	0.27	0.61	0.38	**0.37**	0.29	0.27	0.34	0.37	**0.32**	**0.85**
Cpt1a	0.16	0.39	0.36	0.25	**0.29**	0.34	0.27	0.29	0.21	**0.28**	**0.95**
Cpt2	0.11	0.16	0.27	0.17	**0.18**	0.19	0.23	0.20	0.19	**0.20**	**1.13**
Cyp2e1	5.69	11.48	23.31	12.43	**13.23**	14.40	10.64	7.63	9.53	**10.55**	**0.80**
Cyp7a1	0.18	0.23	0.70	0.09	**0.30**	0.12	0.11	0.11	0.04	**0.10**	**0.32**
Dgat2	0.15	0.27	0.64	0.32	**0.35**	0.34	0.25	0.44	0.34	**0.34**	**0.99**
Fabp1	8.17	10.00	18.78	14.04	**12.75**	16.31	13.16	8.06	16.13	**13.42**	**1.05**
Fabp3	0.00	0.00	0.00	0.00	**0.00**	0.00	0.00	0.00	0.00	**0.00**	**0.80**
Fabp5	0.05	0.04	0.04	0.05	**0.04**	0.05	0.01	0.01	0.06	**0.03**	**0.80**
Fas	0.02	0.03	0.03	0.04	**0.03**	0.04	0.04	0.03	0.03	**0.04**	**1.21**
Fasn	0.51	0.45	0.82	0.87	**0.66**	0.26	0.15	0.19	0.55	**0.29**	**0.43**
Foxa2	0.01	0.03	0.04	0.02	**0.03**	0.01	0.01	0.03	0.02	**0.02**	**0.67**
G6pc	0.17	0.41	0.49	0.29	**0.34**	0.15	0.20	0.25	0.37	**0.24**	**0.72**
G6pdx	0.00	0.00	0.00	0.00	**0.00**	0.01	0.00	0.00	0.01	**0.00**	**1.11**
Gck	0.05	0.11	0.23	0.17	**0.14**	0.14	0.10	0.10	0.14	**0.12**	**0.87**
Gsk3b	0.04	0.06	0.07	0.07	**0.06**	0.05	0.04	0.04	0.04	**0.04**	**0.70**
Gyk	0.03	0.04	0.07	0.06	**0.05**	0.09	0.08	0.08	0.11	**0.09**	**1.70**
Hmgcr	0.07	0.06	0.17	0.26	**0.14**	0.12	0.09	0.07	0.07	**0.09**	**0.62**
Hnf4a	0.19	0.29	0.58	0.43	**0.37**	0.27	0.22	0.51	0.32	**0.33**	**0.89**
Ifng	ND	ND	ND	ND	ND	ND	ND	ND	ND	ND	ND
Igf1	0.80	1.19	2.02	1.53	**1.38**	0.78	1.01	1.03	0.90	**0.93**	**0.67**

Igfbp1	0.03	0.05	0.04	0.01	**0.03**	0.06	0.02	0.10	0.05	**0.06**	**2.01**
Il10	0.00	0.00	0.00	0.00	**0.00**	0.00	0.00	0.00	0.00	**0.00**	**0.81**
Il1b	0.00	0.00	0.00	0.00	**0.00**	0.00	0.01	0.01	0.00	**0.00**	**1.93**
Il6	ND	ND	ND	ND	ND	ND	ND	ND	ND	ND	ND
Insr	0.05	0.07	0.09	0.08	**0.07**	0.05	0.06	0.07	0.06	**0.06**	**0.80**
Irs1	0.03	0.02	0.04	0.03	**0.03**	0.02	0.02	0.04	0.04	**0.03**	**0.93**
Ldlr	0.11	0.13	0.18	0.20	**0.15**	0.15	0.10	0.14	0.13	**0.13**	**0.83**
Lepr	0.00	0.01	0.01	0.00	**0.01**	0.00	0.00	0.00	0.00	**0.00**	**0.56**
Lpl	0.03	0.01	0.01	0.02	**0.02**	0.02	0.02	0.02	0.02	**0.02**	**1.12**
Mapk1	0.11	0.12	0.20	0.16	**0.15**	0.12	0.14	0.12	0.12	**0.12**	**0.84**
Mapk8	0.02	0.03	0.05	0.04	**0.04**	0.03	0.03	0.02	0.03	**0.03**	**0.75**
Mlxipl	0.11	0.08	0.15	0.20	**0.13**	0.07	0.07	0.08	0.11	**0.08**	**0.62**
Mtor	0.01	0.01	0.02	0.02	**0.02**	0.02	0.01	0.01	0.02	**0.02**	**1.08**
Ndufb6	0.07	0.11	0.21	0.15	**0.14**	0.16	0.13	0.17	0.08	**0.13**	**0.98**
Nfkb1	0.01	0.01	0.02	0.02	**0.01**	0.01	0.01	0.02	0.02	**0.02**	**1.13**
Nr1h2	0.02	0.03	0.03	0.04	**0.03**	0.03	0.02	0.03	0.03	**0.03**	**0.94**
Nr1h3	0.05	0.07	0.13	0.10	**0.09**	0.09	0.08	0.12	0.10	**0.10**	**1.10**
Nr1h4	0.08	0.11	0.19	0.14	**0.13**	0.09	0.11	0.09	0.10	**0.10**	**0.76**
Pck2	0.00	0.00	0.00	0.00	**0.00**	0.00	0.00	0.00	0.00	**0.00**	**1.08**
Pdk4	0.00	0.00	0.00	0.00	**0.00**	0.01	0.00	0.00	0.01	**0.00**	**1.44**
Pik3ca	0.02	0.02	0.04	0.03	**0.03**	0.02	0.03	0.02	0.03	**0.03**	**0.92**
Pik3r1	0.01	0.04	0.07	0.04	**0.04**	0.04	0.02	0.02	0.03	**0.03**	**0.68**
Pklr	0.23	0.44	0.65	0.42	**0.44**	0.30	0.22	0.10	0.48	**0.28**	**0.63**
Ppa1	0.13	0.15	0.20	0.18	**0.16**	0.16	0.13	0.19	0.18	**0.17**	**1.01**
Ppara	0.09	0.16	0.22	0.21	**0.17**	0.21	0.19	0.16	0.18	**0.19**	**1.08**
Ppard	0.00	0.00	0.00	0.00	**0.00**	0.00	0.00	0.00	0.00	**0.00**	**1.07**
Pparg	0.01	0.03	0.04	0.03	**0.03**	0.05	0.03	0.01	0.03	**0.03**	**1.02**
Ppargc1a	0.01	0.02	0.02	0.01	**0.01**	0.02	0.01	0.01	0.01	**0.01**	**1.12**
Prkaa1	0.01	0.01	0.01	0.01	**0.01**	0.01	0.01	0.00	0.01	**0.01**	**0.82**
Ptpn1	0.00	0.00	0.01	0.01	**0.01**	0.00	0.00	0.01	0.00	**0.01**	**1.02**
Rbp4	1.79	2.69	4.83	4.20	**3.37**	3.56	2.75	4.38	3.47	**3.54**	**1.05**
Rxra	0.11	0.15	0.21	0.17	**0.16**	0.12	0.10	0.13	0.10	**0.11**	**0.69**
Scd1	4.02	9.06	5.86	4.58	**5.88**	11.77	4.78	3.84	10.93	**7.83**	**1.33**
Serpine1	0.00	0.00	0.00	0.00	**0.00**	0.00	0.00	0.00	0.00	**0.00**	**4.13**
Slc27a5	0.50	0.87	1.74	1.43	**1.14**	1.14	1.29	1.47	1.20	**1.28**	**1.12**
Slc2a1	0.01	0.01	0.01	0.01	**0.01**	0.01	0.01	0.01	0.01	**0.01**	**0.73**
Slc2a2	0.30	0.55	0.82	0.54	**0.55**	0.40	0.33	0.21	0.40	**0.34**	**0.61**
Slc2a4	0.00	0.00	0.00	0.00	**0.00**	0.00	0.00	0.00	0.00	**0.00**	**0.09**
Socs3	0.00	0.00	0.01	0.01	**0.01**	0.00	0.00	0.01	0.00	**0.01**	**1.09**
Srebf1	0.18	0.13	0.52	0.43	**0.32**	0.11	0.12	0.12	0.28	**0.16**	**0.50**
Srebf2	0.01	0.01	0.02	0.03	**0.02**	0.01	0.02	0.02	0.02	**0.02**	**0.99**
Stat3	0.06	0.08	0.08	0.11	**0.08**	0.06	0.06	0.11	0.07	**0.08**	**0.93**
Tnf	ND	ND	ND	ND	ND	ND	ND	ND	ND	ND	ND
Xbp1	0.16	0.21	0.45	0.35	**0.29**	0.14	0.14	0.28	0.21	**0.19**	**0.66**

Supplementary Table 3. The metabolites concentration in lipidomics assay.

	Metabolites	shCrtl		shNogo-B	
	pmol/mg	#1	#2	#1	#2
PL	16:0/18:2 PI	0.1686	0.1293	0.6845	0.7927
	16:0/18:1 PI	0.2822	0.1976	0.5000	0.6419
	16:0/18:0 PI	0.3289	0.3533	0.0402	0.0532
	16:0/20:4 PI	1.3194	0.7485	6.6977	7.3952
	16:0/22:6 PI	0.1654	0.1269	0.5209	0.5935
	18:0/18:2 PI	0.4886	0.4198	1.8062	1.8065
	18:0/18:1 PI	0.2778	0.2593	0.4535	0.5121
	18:0/18:0 PI	0.3245	0.3731	0.0369	0.0402
	18:0/20:4 PI	13.0682	6.9461	65.5814	66.3710
	17:0/20:4 PI	0.0644	0.0333	0.3729	0.3919
	18:0/22:6 PI	0.4268	0.1754	1.7054	1.6290
	16:0/18:2 PE	16.8561	18.5629	84.4961	53.7097
	16:0/18:1 PE	21.7803	22.9341	49.3798	45.0000
	16:0/18:0 PE	20.0126	18.2635	4.8062	4.6371
	17:0/17:0 PE	20.6439	18.1437	4.4419	4.1855
	16:0/20:4 PE	69.4444	76.6467	200.7752	195.9677
	16:0/22:6 PE	154.6717	159.8802	380.6202	371.7742
	18:0/18:2 PE	37.9419	42.6347	89.1473	70.8871
	18:0/18:1 PE	19.5707	22.0958	23.3333	21.0484
	18:0/18:0 PE	13.5732	12.1557	2.3333	2.3468
	18:0/20:4 PE	184.9747	197.6048	406.2016	410.4839
	18:0/22:6 PE	118.6869	117.9641	149.6124	138.7097
	16:0/18:2 PC	258.8384	216.1677	837.2093	780.6452
	16:0/18:1 PC	423.6111	386.2275	945.7364	935.4839
	16:0/18:0 PC	159.7222	138.9222	239.5349	243.5484
	16:0/20:5 PC	31.7551	24.3713	145.7364	116.9355
	16:0/20:4 PC	342.8030	271.2575	751.9380	822.5806
	16:0/20:3 PC	227.9040	182.6347	665.8915	667.7419
	16:0/22:6 PC	340.2778	304.1916	717.0543	712.0968
	18:0/18:2 PC	280.9343	241.3174	708.5271	721.7742
	18:0/18:1 PC	231.6919	213.7725	429.4574	458.8710
	18:0/18:0 PC	56.1869	52.1557	41.9380	47.5806
	18:0/20:4 PC	311.8687	256.8862	600.0000	687.9032
	18:0/20:3 PC	151.5152	108.3832	351.1628	388.7097
	18:0/22:6 PC	219.6970	184.4311	325.5814	342.7419
	16:0 LPI	2.1622	1.2624	1.5903	1.9257
	18:2 LPI	0.2114	0.1411	0.1294	0.1608
	18:1 LPI	0.6471	0.4723	0.7262	1.0502
	18:0 LPI	27.5040	17.1631	15.4361	16.3855

20:4 LPI	1.1383	0.3348	1.8215	1.8373
16:0 LPE	30.6836	14.7518	12.7992	12.3695
18:2 LPE	4.1971	2.2270	2.9209	2.1285
18:1 LPE	9.1256	4.7943	8.8438	11.1647
18:0 LPE	114.6264	60.8511	19.9189	21.4859
20:4 LPE	5.9141	2.8085	4.0162	3.8956
22:6 LPE	17.8060	8.3688	5.1927	4.4779
16:0 LPC	441.9714	187.2340	330.6288	387.5502
18:2 LPC	79.3323	27.6596	90.2637	81.9277
18:1 LPC	131.3196	52.9078	132.6572	158.8353
18:0 LPC	435.6121	198.5816	181.5416	238.9558
20:3 LPC	44.8331	14.3262	27.1805	27.9116
20:4 LPC	85.6916	25.8156	57.4037	72.0884
20:5 LPC	3.0684	0.7645	2.9209	2.4297
22:6 LPC	140.5405	47.5177	40.9736	40.5622
16:0 LPA	0.0054	0.0026	0.0025	0.0025
18:1 LPA	0.0006	0.0004	0.0002	0.0002
18:0 LPA	0.0120	0.0050	0.0020	0.0014
20:4 LPA	0.0099	0.0036	0.0553	0.0686
22:6 LPA	0.0480	0.0184	0.0681	0.0499
AA	17.1125	15.1207	1.2068	1.6165
DHA	14.4798	15.7586	1.2727	1.5024
18:0 S1P	0.0507	0.0574	0.0385	0.0442
17:1 S1P	0.0009	0.0004	0.0089	0.0070
16:0/22:6 PA	0.0053	0.0091	0.0041	0.0016
18:0/20:4 PA	0.0231	0.0193	0.0140	0.0144
18:0/22:6 PA	0.0588	0.0450	0.0815	0.0678
16:0 LPG	0.1817	0.1076	0.0405	0.0572
18:2 LPG	0.1361	0.0616	0.0100	0.0123
18:1 LPG	0.1155	0.0450	0.0289	0.0422
18:0 LPG	0.1255	0.0807	0.0098	0.0154
20:4 LPG	0.0132	0.0062	0.0003	0.0015
22:6 LPG	0.0431	0.0345	0.0024	0.0033
16:0/18:2 PG	0.0229	0.0164	0.0111	0.0139
16:0/18:1 PG	0.0669	0.0559	0.0941	0.0895
16:0/20:4 PG	0.0270	0.0278	0.0041	0.0058
18:0/18:2 PG	0.0201	0.0144	0.0134	0.0177
18:0/18:1 PG	0.0121	0.0084	0.0033	0.0043
18:0/22:6 PG	0.0588	0.0450	0.0815	0.0678
16:0 LPS	0.0352	0.0178	0.1442	0.2029
18:2 LPS	0.0055	0.0029	0.0153	0.0162
18:1 LPS	0.0197	0.0096	0.0791	0.1306
18:0 LPS	0.4798	0.2776	0.9572	1.3439

	20:4 LPS	0.0461	0.0171	0.4046	0.4960
	22:6 LPS	0.0431	0.0345	0.0024	0.0033
	16:0/18:2 PS	0.0046	0.0049	0.0301	0.0309
	16:0/18:1 PS	0.0195	0.0174	0.0627	0.0756
	16:0/20:4 PS	0.0592	0.0284	1.4403	1.6323
	16:0/22:6 PS	0.0459	0.0212	1.6649	0.9810
	18:0/18:2 PS	0.0584	0.0412	0.3601	0.3994
	18:0/18:1 PS	0.1437	0.1071	0.6292	0.5737
	18:0/18:0 PS	0.0282	0.0214	0.0661	0.0645
	18:0/20:4 PS	0.5159	0.2569	7.3440	8.3360
	18:0/22:6 PS	0.2144	0.1112	3.8324	2.4406
	C12:0	543.9673	757.3696	958.2689	712.9630
	C14:0	1014.3149	1412.6984	2071.0974	2114.1975
	C15:0	45.8078	58.2766	103.2457	91.5123
	C16:0	6462.1677	7210.8844	14064.9150	20061.7284
	C16:1	2862.9857	3356.0091	4049.4590	5787.0370
	C16:2	18.4867	26.7574	23.1839	41.3580
	C18:0	4314.9284	4716.5533	1622.8748	1697.5309
	C18:1	6666.6667	7687.0748	13431.2210	20216.0494
	C18:2	3742.3313	4081.6327	3091.1901	3410.4938
	C18:3	179.7546	253.9683	265.8423	266.9753
FA	C20:1	306.7485	437.6417	420.4019	541.6667
	C20:2	160.9407	189.1156	131.3756	195.9877
	C20:3	881.3906	943.3107	400.3091	486.1111
	C20:4	4233.1288	4467.1202	1375.5796	1790.1235
	C22:1	22.4949	25.1701	42.1947	50.1543
	C22:3	30.0613	36.0544	27.8207	38.5802
	C22:4	81.3906	95.6916	49.6136	81.6358
	C22:5	323.1084	405.8957	137.2488	174.3827
	C22:6	1770.9611	2185.9410	621.3292	679.0123
	C24:6	15.2761	23.5828	24.8841	20.3704
	C24:8	65.0307	71.8821	69.3972	105.5556
	18:1/18:2 DG	50.5128	89.4009	61.5385	100.0000
	18:1/18:1 DG	375.8974	520.7373	384.0237	584.7953
	16:0/16:0/16:0	11.0256	9.1705	7.1598	22.1053
	16:0/14:0/18:1	269.7436	262.6728	353.8462	378.9474
	16:1/16:0/16:1	152.3077	141.9355	213.6095	215.2047
TG	16:1/14:0/18:1	106.6667	115.2074	175.1479	154.3860
	18:1/12:0/18:1	42.6667	58.0645	85.2071	68.4211
	18:2/16:1/14:0	12.0513	12.5806	25.1479	17.8363
	18:2/18:1/12:0	9.3333	12.4885	24.9112	16.1988
	16:0/18:0/16:0	1.0051	1.0968	0.7692	1.7895
	16:0/18:1/16:0	315.3846	340.0922	344.9704	527.4854

18:1/16:0/16:1	477.9487	451.1521	534.9112	719.2982
18:1/14:0/18:1	114.3590	123.5023	166.2722	171.9298
16:1/18:1/16:1	162.0513	164.9770	220.1183	294.1520
18:2/16:1/16:0	58.4615	60.3687	101.1834	107.6023
18:2/14:0/18:1	79.4872	85.7143	135.5030	143.8596
16:1/18:2/16:1	18.4103	17.5576	35.6805	33.9181
18:2/14:0/18:2	4.0154	3.6129	11.5976	6.2573
18:3/14:0/18:2	0.4415	0.5161	1.5266	0.9240
18:3/16:1/16:1	1.6872	1.1659	2.9941	1.9298
18:1/16:0/18:0	6.4615	7.4194	7.3964	9.0643
18:1/16:0/18:1	769.2308	824.8848	905.3254	1175.4386
18:1/16:1/18:1	128.7179	146.0829	142.6036	238.0117
18:1/18:2/16:0	148.2051	163.1336	211.2426	261.4035
16:1/18:1/18:2	26.7179	30.2765	47.0414	60.2339
18:2/16:0/18:2	19.8974	19.3088	31.6568	27.9532
18:2/16:1/18:2	3.8154	4.5346	10.5917	8.2456
18:3/16:0/18:2	4.6103	4.8848	10.0000	7.5439
18:3/16:1/18:2	0.6154	0.5438	1.8107	0.8830
20:4/14:0/18:2	1.5436	1.5253	3.1953	2.4912
18:1/18:0/18:1	3.5538	4.7465	4.9349	5.9649
20:1/16:0/18:1	12.3077	14.7926	15.0296	23.6842
18:1/18:1/18:1	156.4103	197.2350	192.8994	351.4620
18:2/18:0/18:1	3.5897	4.8848	5.6746	6.9591
18:1/18:2/18:1	16.7692	20.5991	24.4970	36.0234
20:3/16:0/18:1	3.6513	4.0553	5.9053	6.0234
20:4/18:1/16:0	12.5128	12.5346	16.3314	22.5146
20:4/16:0/18:2	5.8462	6.4977	11.8935	11.7544
20:4/16:1/18:2	0.7231	0.7143	1.5799	1.4269
20:3/16:2/18:2	1.3641	1.4608	3.7278	2.0351

Supplementary Table 4. The shRNA and siRNA sequences used in this study.

shRNA/siRNA	Sequence(5’-3’)
shCtrl (human)	GTTCTCCGAACGTGTCACGT
shNogo-B-1	GTCCCTGGATTGAAGCGCAA
shNogo-B-2	GATCGTTGTTAGATCTTTA
shCtrl (mouse)	GTAGCTATACGAACGTAGT
shNogo-B	GTTGCCATATCAGAGGAAT
shATG5-1	GCAACTCTGGATGGGATTG
shATG5-2	GCAGAACCATACTATTTGCTT
shNgBR-1	CGGTCAATAAGTTGTAATCTTG
shNgBR-2	AAGGAAATACATAGACCTACA
siATG5-1	AAUUCGUCCAAACCACACAUCUCGA
siATG5-2	GGAAUAUCCUGCAGAAGAATT
siCEBPb-1	GGCCCUGAGUAAUCGCUUATT
siCEBPb-2	GAAGACCGUGGACAAGCACTT

Supplementary Table 5. The primers used in this study.

Primer name	Species	Sequence(5'-3')	Application
Nogo-B-F	mouse	GGCTCAGTGGTTGTTGACCT	qRT-PCR
Nogo-B-R	mouse	TCAGAGACAGCAGCAGGAATAA	qRT-PCR
Gapdh-F	mouse	AATGGATTTGGACGCATTGGT	qRT-PCR
Gapdh-R	mouse	TTTGCACTGGTACGTGTTGAT	qRT-PCR
Cd36-F	mouse	CTGGGACCATTGGTGATGAAA	qRT-PCR
Cd36-R	mouse	CACCACTCCAATCCCAAGTAAG	qRT-PCR
Ctgf-F	mouse	GGGCCTCTTCTGCGATTTC	qRT-PCR
Ctgf-R	mouse	ATCCAGGCAAGTGCATTGGTA	qRT-PCR
Cyr61-F	mouse	CTGCGCTAAACAACTCAACGA	qRT-PCR
Cyr61-R	mouse	GCAGATCCCTTTCAGAGCGG	qRT-PCR
Sra-F	mouse	TGAAGACGAGGACATGCCATC	qRT-PCR
Sra-R	mouse	GAGGTACAAAATCCGCACTGA	qRT-PCR
Sr-b1-F	mouse	TTTGGAGTGGTAGTAAAAAGGGC	qRT-PCR
Sr-b1-R	mouse	TGACATCAGGGACTCAGAGTAG	qRT-PCR
Srec-F	mouse	TGGGACTAGAGCTGGTGTTCT	qRT-PCR
Srec-R	mouse	CAGATGGGGATGGTGCATTCT	qRT-PCR
Lox1-F	mouse	CAAGATGAAGCCTGCGAATGA	qRT-PCR
Lox1-R	mouse	ACCTGGCGTAATTGTGTCCAC	qRT-PCR
Tnf-α-F	mouse	TCTTCTCATTCCTGCTTGTGG	qRT-PCR
Tnf-α-R	mouse	GGTCTGGGCCATAGAACTGA	qRT-PCR
Il6-F	mouse	GCTACCAAACTGGATATAATCAGGA	qRT-PCR
Il6-R	mouse	CCAGGTAGCTATGGTACTCCAGAA	qRT-PCR
Nogo-B-F	human	TCGGCTCAGTGGTTGTTGAC	qRT-PCR
Nogo-B-R	human	GTCAGTGATAGCAGCAGGAATA	qRT-PCR
CTGF-F	human	CCAATGACAACGCCTCCTG	qRT-PCR
CTGF-R	human	TGGTGCAGCCAGAAAGCTC	qRT-PCR
CYR61-F	human	CTCGCCTTAGTCGTCACCC	qRT-PCR
CYR61-R	human	CGCCGAAGTTGCATTCCAG	qRT-PCR
CEBPβ-F	human	TCCAAACCAACCGCACAT	qRT-PCR
CEBPβ-R	human	AGAGGGAGAAGCAGAGAGTTTA	qRT-PCR
GAPDH-F	human	CATCACCATCTTCCAGGAGCG	qRT-PCR
GAPDH-R	human	TGACCTTGCCCACAGCCTTG	qRT-PCR
PNPLA2-F	human	AACACCAGCATCCAGTTCA	qRT-PCR
PNPLA2-R	human	TATCCCTGCTTGCACATCTC	qRT-PCR
LIPE-F	human	ACCACAGCAATCACCTTACAT	qRT-PCR
LIPE-F	human	GCTGGCTCCTGTTGAGTTATAG	qRT-PCR
CEBPβ-1-F	human	TACGGCACTTCCTCTCTCTTA	ChIP-PCR
CEBPβ-1-R	human	ACTCACGGTTACCCAAAGTG	ChIP-PCR

Supplementary Table 6. The antibodies used in this study.

**Antibody**	**Company**	**Item NO.**	**Dilution Fold**
anti-Nogo-B	R&D	AF6034	1:1000
anti-GAPDH	Beyotime	AF0006	1:1000
anti-CD36	Abcam	Ab33625	1:1000
anti-CEBPβ	Abcam	ab15049	1:300
anti-ATG5	CST	12994	1:1000
anti-ATG7	CST	8558	1:1000
anti-Nogo B receptor	Abcam	ab168351	1:1000
anti-LC3	Sigma	L7543	1:2000
anti-p62	CST	39749	1:1000
anti-p-YAP	CST	13008	1:500
anti-YAP	CST	14074	1:500
anti-oxLDL	Biorbyt	orb10973	1:500
anti-PLIN2	Abcam	ab108323	1:1000
anti-RAB7	CST	9367	1:1000
anti-Calnexin	CST	2679	1:1000
anti-Histone H3	CST	9715	1:1000
Anti-rabbit IgG (H+L), F(ab')2 Fragment (Alexa Fluor® 488 Conjugate)	CST	4412	1:1000
Anti-rabbit IgG (H+L), F(ab')2 Fragment (Alexa Fluor® 555 Conjugate)	CST	4413	1:1000
Alexa Fluor® 647 Conjugate Goat Anti-rabbit IgG (H+L)	Beyotime	A0468	1:1000
HRP-labeled Goat Anti-Rabbit IgG(H+L)	Beyotime	A0208	1:1000
HRP-labeled Goat Anti-Mouse IgG(H+L)	Beyotime	A0216	1:1000

Supplementary Table 7. The plasmids used in this study.

Plasmids	Source
pCDH-CMV-MCS-EF1-Puro	System Biosciences
pCDH-NogoB	This paper
pCDH-NogoB-d38	This paper
pCDH-NogoB-Flag	This paper
pLKO.1 hygro	Addgene
pLKO.1 shCtrl	This paper
pLKO.1 shNogoB 1#	This paper
pLKO.1 shNogoB 2#	This paper
pLKO.1 shATG5 1#	This paper
pLKO.1 shATG5 2#	This paper
pLKO.1 shNgBR 1#	This paper
pLKO.1 shNgBR 2#	This paper
pLL3.7	Addgene
pLL3.7 shctrl	This paper
pLL3.7 shNogoB	This paper
pBABE-puro mCherry-EGFP-LC3B	Addgene
pGL4.20	Promega
pGL4.20-4×TBD	This paper
pRL-CMV	Promega
psPAX2	Addgene
pMD2.G	Addgene
pEGFP-LC3B	This paper
pEGFP-Nogo-B	This paper
pEGFP-RAB7	This paper

Supplementary Table 8. Clinical pathological information of the NAFLD-associated HCC patients.

Patient no.	Sex	Age	Hep B	Hep C	Fatty liver	Diabetes	Hypertension	Dyslipidemia
1	male	72	negative	negative	Yes	Yes	Yes	Yes
2	female	36	negative	negative	No	No	Yes	No
3	male	58	negative	negative	No	Yes	Yes	No
4	male	64	negative	negative	Yes	Yes	Yes	No
5	male	78	negative	negative	Yes	No	No	No
6	male	69	negative	negative	Yes	Yes	Yes	No
7	male	75	negative	negative	Yes	No	No	No
8	male	71	negative	negative	No	Yes	Yes	No
9	male	58	negative	negative	No	Yes	Yes	Yes
10	female	74	negative	negative	No	Yes	No	No
11	male	74	negative	negative	Yes	No	Yes	No
12	male	60	negative	negative	Yes	Yes	Yes	No
13	male	59	negative	negative	Yes	No	Yes	No
14	male	55	negative	negative	Yes	Yes	No	No
15	male	62	negative	negative	Yes	No	No	No
16	male	74	negative	negative	Yes	Yes	Yes	Yes

Supplementary Table 9. Univariate and Multivariate Analyses of Prognostic Factors in HCC Cohort (n =360).

Variable	Hazard Ration	Lower	Higher	P value	Patient Number
CD36(high *vs* low)	0.95	0.61	1.5	0.83	161:161
CYR61(high *vs* low)	0.85	0.54	1.3	0.48	161:161
Nogo-B(high *vs* low)	1.5	0.99	2.4	0.05	161:161
Gender(male *vs* female)	0.82	0.52	1.3	0.41	218:104
age(>50 *vs* <=50)	1.6	0.8	3	0.19	258:64
alcohol(yes *vs* no)	0.864	0.508	1.469	0.59	107:191
HBV(yes *vs* no)	0.508	0.197	1.308	0.16	155:15
Tumor stage(T3/4 *vs* T1/2)	2.083	1.307	3.319	0.002	81:240
cirrhosis(yes *vs* no)	0.781	0.404	1.512	0.46	58:121
Vascular invasion(yes *vs* no)	1.398	0.839	2.33	0.2	94:176
AFP(>500 *vs* <=500 ng/mL)	0.94	0.529	1.67	0.83	188:54
inflammation(yes *vs* no)	1.054	0.6	1.852	0.85	105:96
Multivariate analysis					
Nogo-B(high *vs* low)	1.621	1.039	2.529	0.03	321
Tumor stage(T3/4 *vs* T1/2)	2.164	1.357	3.451	0.001	321

Supplementary Table 10. Proportionality assumption in univariate analysis

Variable	rho	chisq	P Value
CD36(high *vs* low)	-0.182	2.796	0.09
CYR61(high *vs* low)	-0.057	0.256	0.61
Nogo-B(high *vs* low)	-0.207	3.34	0.07
Gender(male *vs* female)	-0.075	0.449	0.5
age(>50 *vs* <=50)	-0.056	0.248	0.618
alcohol(yes *vs* no)	0.149	1.5	0.22
HBV(yes *vs* no)	-0.0525	0.119	0.73
Tumor stage(T3/4 *vs* T1/2)	0.197	3.02	0.08
cirrhosis(yes *vs* no)	-0.255	3.1	0.08
Vascular invasion(yes *vs* no)	-0.021	0.029	0.86
AFP(>500 *vs* <=500)	0.119	0.947	0.33
inflammation(yes *vs* no)	-0.105	0.553	0.46

Supplementary Table 11. Proportionality assumption in multivariate analysis

Variable	r ho	chisq	P Value
Nogo-B	-0.17	2.19	0.14
Tumor stage	0.2	3.1	0.08
GLOBAL	NA	5.72	0.06
