# Supplementary material for: ER-residential Nogo-B accelerates NAFLD-associated HCC mediated by metabolic reprogramming of oxLDL lipophagy
Source: Nat Commun. 2019 Jul 29;10:3391. doi: 10.1038/s41467-019-11274-x (PMC6662851; doi:10.1038/s41467-019-11274-x)
Supplement: Supplementary file 2 — Reporting Summary [file 41467_2019_11274_MOESM2_ESM.pdf]

nature research

Corresponding author(s): Alfred S.L. Cheng, Pengyuan Yang

Last updated by author(s): Jun 6, 2019

Reporting Summary

Nature Research wishes to improve the reproducibility of the work that we publish. This form provides structure for consistency and transparency in reporting. For further information on Nature Research policies, see Authors & Referees and the Editorial Policy Checklist.

Statistics

For all statistical analyses, confirm that the following items are present in the figure legend, table legend, main text, or Methods section.

n/a	Confirmed	
☐	☒	The exact sample size (*n*) for each experimental group/condition, given as a discrete number and unit of measurement
☐	☒	A statement on whether measurements were taken from distinct samples or whether the same sample was measured repeatedly
☐	☒	The statistical test(s) used AND whether they are one- or two-sided<br>*Only common tests should be described solely by name; describe more complex techniques in the Methods section.*
☒	☐	A description of all covariates tested
☐	☒	A description of any assumptions or corrections, such as tests of normality and adjustment for multiple comparisons
☐	☒	A full description of the statistical parameters including central tendency (e.g. means) or other basic estimates (e.g. regression coefficient) AND variation (e.g. standard deviation) or associated estimates of uncertainty (e.g. confidence intervals)
☐	☒	For null hypothesis testing, the test statistic (e.g. *F*, *t*, *r*) with confidence intervals, effect sizes, degrees of freedom and *P* value noted<br>*Give P values as exact values whenever suitable.*
☒	☐	For Bayesian analysis, information on the choice of priors and Markov chain Monte Carlo settings
☒	☐	For hierarchical and complex designs, identification of the appropriate level for tests and full reporting of outcomes
☐	☒	Estimates of effect sizes (e.g. Cohen's *d*, Pearson's *r*), indicating how they were calculated

Our web collection on statistics for biologists contains articles on many of the points above.

Software and code

Policy information about availability of computer code

Data collection	The TCGA data reference in this study are available in the cBioPortal for Cancer Genomics website and Oncomine website.
Data analysis	GraphPad Prism 5 was used for data analysis.

For manuscripts utilizing custom algorithms or software that are central to the research but not yet described in published literature, software must be made available to editors/reviewers. We strongly encourage code deposition in a community repository (e.g. GitHub). See the Nature Research guidelines for submitting code & software for further information.

Data

Policy information about availability of data

All manuscripts must include a data availability statement. This statement should provide the following information, where applicable:

- Accession codes, unique identifiers, or web links for publicly available datasets
- A list of figures that have associated raw data
- A description of any restrictions on data availability

The TCGA data reference in this study are available in the cBioPortal for Cancer Genomics website and Oncomine website. The authors declare that all the other data supporting the findings of this study are available within the article and its Supplementary Information files and from the corresponding author upon reasonable request. The source data underlying all bar charts are provided as a Source Data file.

Field-specific reporting

Please select the one below that is the best fit for your research. If you are not sure, read the appropriate sections before making your selection.

☒ Life sciences ☐ Behavioural & social sciences ☐ Ecological, evolutionary & environmental sciences

For a reference copy of the document with all sections, see nature.com/documents/nr-reporting-summary-flat.pdf

Life sciences study design

All studies must disclose on these points even when the disclosure is negative.

Sample size	Sample sizes were chosen to satisfy statistical power based on previous experience and knowledge. For animal studies, we performed power analyses using a web-based tool at www.biomath.info.
Data exclusions	Experiments were excluded that were technically invalid.
Replication	The replication of all experiments was successful.
Randomization	To establish the NASH-associated HCC mouse model, mice were randomly assigned to LFD or high-fat, high-carbohydrate (HFHC) diet.
Blinding	Blinding in in vivo experiments was not done during experimentation, but the labels were covered during data analysis.

Reporting for specific materials, systems and methods

We require information from authors about some types of materials, experimental systems and methods used in many studies. Here, indicate whether each material, system or method listed is relevant to your study. If you are not sure if a list item applies to your research, read the appropriate section before selecting a response.

Materials & experimental systems

n/a	Involved in the study
☐	☒ Antibodies
☐	☒ Eukaryotic cell lines
☒	☐ Palaeontology
☐	☒ Animals and other organisms
☐	☒ Human research participants
☒	☐ Clinical data

Methods

n/a	Involved in the study
☒	☐ ChIP-seq
☒	☐ Flow cytometry
☒	☐ MRI-based neuroimaging

Antibodies

Antibodies used	anti-Nogo-B (R&D, AF6034), anti-GAPDH (Beyotime, AF0006), anti-CD36 (Abcam, Ab33625) , anti-CEBPβ (Abcam, ab15049), anti-ATG5 (CST, 12994), anti-ATG7 (CST, 8558) anti-Nogo-B receptor (Abcam, ab168351) , anti-LC3 (Sigma, L7543), anti-p62 (CST, 39749), anti-p-YAP (CST, 13008), anti-YAP (CST, 14074), anti-oxLDL (Biorbyt, orb10973), anti-PLIN2 (Abcam, ab108323), anti-RAB7 (CST, 9367), anti-Calnexin (CST, 2679), anti-Histone H3 (CST, 9715).
Validation	anti-Nogo-B (manufacturer's website), anti-GAPDH (PMID 29870774), anti-CD36 (PMID 30717785), anti-CEBPβ (PMID 28114367), anti-ATG5 (PMID 26609472), anti-ATG7 (PMID 26607902) anti-Nogo-B receptor (PMID 26755743), anti-LC3 (PMID 30348528), anti-p62 (PMID 30323296), anti-p-YAP (PMID 27836738), anti-YAP (PMID 30745823). anti-oxLDL (PMID 27010927), anti-PLIN2 (PMID 27942596), anti-RAB7 (PMID 26820848), anti-Calnexin (PMID 29137221), anti-Histone H3 (PMID 28112184).

Eukaryotic cell lines

Policy information about cell lines

Cell line source(s)	Hep3B, HepG2, PLC5 SK-Hep1 and RAW264.7 cells were obtained from the American Type Culture Collection (ATCC, Manassas, Virginia, USA). Huh7 cells were obtained from the Japanese Collection of Research Bioresources (JCRB, Tokyo, Japan). LO2 and BEL-7404 were obtained from the Cellosaurus. SMMC-7721 cell line was purchased from the Shanghai Cell Bank Type Culture Collection Committee (CBTCCC, Shanghai, China). MHCC97L and MHCC97H cells were gifts from Fudan University (Dr Zhaoyou Tang) of Shanghai. HepG2.2.15 cell line was a gift from Eastern Hepatobiliary Surgery Hospital (Dr. Hongyang Wang) of Shanghai.
Authentication	Cell lines were authenticated by short tandem repeats (STR) profiling.

Mycoplasma contamination	Cell lines were free of mycoplasma.
Commonly misidentified lines (See ICLAC register)	BEL-7404 and SMMC-7721 were used in this study. BEL-7404 was used once to confirm interaction between Nogo-B and ATG5. SMMC-7721 was used in Nogo-B over-expression experiments because Nogo-B expressed lowly in this cell line.

Animals and other organisms

Policy information about studies involving animals; ARRIVE guidelines recommended for reporting animal research

Laboratory animals	Female 6-week-old Nude mice and male 14-day-old C57BL/6 mice.
Wild animals	Nil
Field-collected samples	Nil
Ethics oversight	The ethics committee of Institute of Biophysics, Chinese Academy of Sciences.

Note that full information on the approval of the study protocol must also be provided in the manuscript.

Human research participants

Policy information about studies involving human research participants

Population characteristics	All patients were defined as characteristic of metabolic syndrome, including diabetes, hypertension, dyslipidemia or fatty liver changes, but no record of virus infection nor alcoholic intake. The detailed information was described in Supplementary Table 8.
Recruitment	The patients were informed, and they signed consent forms acknowledging the use of their resected tissues for research purposes.
Ethics oversight	The study was approved by the Joint CUHK-NTEC Clinical Research Ethics Committee.

Note that full information on the approval of the study protocol must also be provided in the manuscript.
